# Both SUMOylation and ubiquitination of TFE3 fusion protein regulated by androgen receptor are the potential target in the therapy of Xp11.2 translocation renal cell carcinoma

**DOI:** 10.1002/ctm2.797

**Published:** 2022-04-22

**Authors:** Ning Liu, Yi Chen, Lei Yang, Qiancheng Shi, Yanwen Lu, Wenliang Ma, Xiaodong Han, Hongqian Guo, Dongmei Li,, Weidong Gan,

**Affiliations:** ^1^ Department of Urology Affiliated Drum Tower Hospital of Medical School of Nanjing University Nanjing Jiangsu China; ^2^ Immunology and Reproduction Biology Laboratory & State Key Laboratory of Analytical Chemistry for Life Science Medical School Nanjing University Nanjing Jiangsu China; ^3^ Jiangsu Key Laboratory of Molecular Medicine Nanjing University Nanjing Jiangsu China; ^4^ Department of Urology Affiliated Sir Run Run Hospital Nanjing Medical University Nanjing Jiangsu China

**Keywords:** progression, SUMOylation, TFE3, translocation RCC, ubiquitination

## Abstract

**Background:**

The aggressiveness of renal cell carcinoma (RCC) associated with Xp11.2 translocation/TFE3 gene fusion (Xp11.2 translocation RCC [Xp11.2 tRCC]) is age‐dependent, which is similar to the overall trend of reproductive endocrine hormones. Therefore, this study focused on the effect and potential mechanism of androgen and androgen receptor (AR) on the progression of Xp11.2 tRCC.

**Methods:**

The effects of androgen and AR on the proliferation and migration of Xp11.2 tRCC cells were first evaluated utilising Xp11.2 tRCC cell lines and tissues. Because Transcription factor enhancer 3 (TFE3) fusion proteins play a key role in Xp11.2 tRCC, we focused on the regulatory role of AR and TFE3 expression and transcriptional activity.

**Results:**

When Xp11.2 tRCC cells were treated with dihydrotestosterone, increased cell proliferation, invasion and migration were observed. Compared with clear cell RCC, the positive rate of AR in Xp11.2 tRCC tissues was higher, and its expression was negatively associated with the progression‐free survival of Xp11.2 tRCC. Further studies revealed that AR could positively regulate the transcriptional activity of TFE3 fusion proteins by small ubiquitin‐related modifier (SUMO)‐specific protease 1, inducing the deSUMOylation of TFE3 fusion. On the other hand, UCHL1 negatively regulated by AR plays a role in the deubiquitination degradation of the PRCC‐TFE3 fusion protein. Therefore, the combination of the AR inhibitor MDV3100 and the UCHL1 inhibitor 6RK73 was effective in delaying the progression of Xp11.2 tRCC, especially PRCC‐TFE3 tRCC.

**Conclusions:**

Androgen and AR function as facilitators in Xp11.2 tRCC progression and may be a novel therapeutic target for Xp11.2 tRCC.

The combined use of AR antagonist MDV3100 and UCHL1 inhibitor 6RK73 increased both the SUMOylation and ubiquitination of the PRCC‐TFE3 fusion protein

## Graphical abstract

Androgen receptor (AR) regulates the SUMO modification of the TFE3 fusion proteins by small ubiquitin‐related modifier‐specific protease 1.

AR affects the ubiquitination of the PRCC‐TFE3 fusion protein by regulating UCHL1 negatively.

Targeting AR and UCHL1 may serve as a novel target for Xp11.2 translocation renal cell carcinoma therapy.

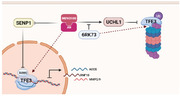

## 1 BACKGROUND

Xp11.2 translocation renal cell carcinoma (Xp11.2 tRCC) is a distinctive tumour that was categorised into the microphthalmia transcription factor (MiT) family tRCC in 2016 by the World Health Organisation.^1^ According to the reciprocal gene, more than 10 fusion subtypes have been reported, including *PRCC‐TFE3, NONO‐TFE3, PSF‐TFE3, RBM10‐TFE3* and others.^2^ It is acknowledged that most Xp11.2 tRCCs share invasive and aggressive progression as one of the most malignant RCC types.^3^, ^4^ Interestingly, early studies linked Xp11.2 tRCC to the age‐dependent prognostic difference, with more aggressive malignancies found in adults.^5^, ^6^, ^7^ Xp11.2 tRCC patients aged over 14 years are more likely to exhibit advanced stage or nuclear pleomorphism, while those younger than 14 years tend to be indolent and progress slowly.^5^ However, the detailed mechanisms underlying this age‐dependent prognostic difference in Xp11.2 tRCC remain unclear.

Wild‐type Transcription factor enhancer 3 (TFE3), a member of the MiT family, mainly regulates energy metabolism by promoting the expression of lysosomal genes in response to nutrient stress.^8^, ^9^ In addition, MiT/TFE proteins are involved in many cellular processes, including innate immunity and inflammation, energy metabolism, nutrient sensing and other processes.^9^, ^10^, ^11^, ^12^, ^13^, ^14^, ^15^, ^16^ In a considerable fraction of human melanomas, Microphthalmia‐associated transcription factor (MITF) is regarded as an oncogene and plays a key role in tumour progression.^17^, ^18^ Studies have demonstrated that androgen receptor (AR) is important in promoting the metastasis of melanoma by targeting MITF.^19^ Furthermore, a study that reviewed 403 genetically confirmed Xp11.2 tRCCs found that the clinically aggressive behaviour tendency of lifetime variation was consistent with that of reproductive endocrine hormones,^6^ suggesting that sex hormones play a positive role in the occurrence and development of Xp11.2 tRCC. However, the potential roles of AR in the progression of this tumour remain to be determined.

The overexpression of the TFE3 fusion protein induced by the rearrangement of the TFE3 gene is the most prominent characteristic of all subtypes of Xp11.2 tRCC.^20^ Structurally, wild‐type TFE3 consists of a transactivating zone, a DNA contact and binding domain and a basic helix‐loop‐helix leucine zipper (bHLH‐Zip) motif, which is responsible for the recognition of transcription initiation or E‐box sites in the genome.^10^, ^21^ TFE3 fusion proteins with the DNA binding domain and the bHLH‐Zip motif of TFE3 have the potential capacity to transcriptionally activate target genes as transcription factors in Xp11.2 tRCC.^3^, ^4^ Our previous study demonstrated that overexpressed TFE3 fusion proteins with strong nuclear retention escaped the control of the mammalian target of rapamycin (mTOR) pathway.^22^ Therefore, controlling the expression, transcriptional activity and subcellular location of TFE3 might be three key points to control the progression of Xp11.2 tRCC.

Posttranscriptional regulation of MiT/TFE plays a crucial role in the adaptation of cell homeostasis to environmental cues, including phosphorylation, acetylation, SUMOylation (where SUMO is small ubiquitin‐related modifier), oxidation and ubiquitination.^13^, ^23^, ^24^ Numerous studies have confirmed that phosphorylation at S321 by mTOR mainly regulates the subcellular location of the wild‐type TFE3 protein instead of the TFE3 fusion proteins.^8^, ^21^, ^24^, ^25^, ^26^ SUMOylation is one of the posttranslational modifications of proteins by covalently conjugating SUMOs to lysine residues of target proteins,^27^, ^28^ which participate in many cellular pathways by regulating the subcellular location, dimerisation, DNA blinding or transcriptional activity of target proteins.^27^, ^28^, ^29^, ^30^ SUMO‐1 is an 11 kDa protein and acts as the dominant SUMO type, and MITF was identified as one of the target proteins of SUMO1.^31^, ^32^ Several studies on melanoma and RCC identified accelerated tumour progression and poor clinical outcome by improving the transcriptional activity of MITF upon mutating its SUMOylation residues.^33^, ^34^ Although the regulation of transcriptional activity by SUMOylated MITF and TFE3 has been established and multiple studies have now established the regulation loop between AR and SUMOylation,^35^, ^36^, ^37^ the association between TFE3 fusion proteins and SUMOylation in Xp11.2 tRCC cells and the relationship between AR and SUMOylation of TFE3 fusion proteins remain unknown.

In this study, we aimed to explore the relevance of androgen/AR to the progression of Xp11.2 tRCC and further reveal the detailed mechanism by which AR affects the progression of Xp11.2 tRCC and then explore interventions for Xp11.2 tRCC by targeting AR. Such investigation is important for clarifying the molecular mechanism for the age dependence of Xp11.2 tRCC progression, thus allowing us to provide a theoretical basis for the effective therapeutic target of this rare tumour.

## 2 METHODS

### 2.1 Clinical samples

From 2013 to 2020, 46 cases of Xp11.2 tRCC patients with tumour issues were identified by TFE3 fluorescence in situ hybridisation at the Affiliated Drum Tower Hospital of Medical School of Nanjing University, and 13 cases of clear cell RCC (ccRCC) were randomly selected as controls. All patients provided signed informed consent for the use of their tissues for scientific research. The current study was approved by the Institutional Review Board.

### 2.2 Cell culture and transfection

The cell lines HEK293T, 786–O, HK‐2 and ACHN were purchased from ATCC (CRL3216, CRL‐1932, CRL2190). UOK120 and UOK109, human carcinoma cell lines characterised by *PRCC‐TFE3* translocation and *NONO* translocation, respectively, were gifts from the Tumour Cell Line Repository of the National Cancer Institute. All cells were cultured in 90% Dulbecco's Modified Eagle Medium (Gibcond, 10569010) + 10% fetal bovine serum (Gibco, 16140063) + 1% penicillin/streptomycin (Invitrogen, 15070063). In the androgen‐deprivation experiment, 10% fetal bovine serum was replaced with 10% charcoal‐stripped media (CSM). For every experiment, at least three different donors were used. All human cell lines were cultured mycoplasma‐free and authenticated by short tandem repeat profiles (or single nucleotide polymorphism) profiling. For treatment, cells were exposed to dihydrotestosterone (DHT) (10 nM, Sigma–Aldrich, D‐073), MDV3100 (10 μM, MCE, HY‐70002B), ASC‐J9 (5 μM, MCE, HY‐15194), 6RK73 (5 μM MCE, HY‐133118), MG‐132 (50 μM, MCE, HY‐13259) or combinations for either 24, 48 or 72 h.

To generate AR‐overexpressing, overexpressing, SUMO‐specific protease 1 (SENP1) AR knocked down, SENP1 knocked down or UCHL1 knocked down stable cell populations, UOK109, UOK120, 786‐O or HEK‐293T cells were infected with pCDH‐AR/pCDH‐Vec, pCDH‐SENP1/pCDH‐Vec, pLV‐shAR‐1/pLV‐scr (CACCAATGTCAACTCCAGGAT), pLV‐shAR‐2/pLV‐scr (CACCAATGTCAACTCCAGGAT), pLV‐shAR‐3/pLV‐scr (GAGCGTGGACTTTCCGGAAAT), pLV‐shSENP1/pLV‐scr (CCGAAAGACCTCAAGTGGATT) or pLV‐shUCHL1/pLV‐scr (CGGGTAGATGACAAGGTGAAT) by using the Lipofectamine 2000 reagent (Invitrogen, 11668019). The psPAX2 packaging plasmid pMD2. G envelope plasmid and the transfer plasmid were transfected into HEK293T cells to produce lentiviruses. Virus supernatants were collected 48 and 72 h after transfection.

### 2.3 Plasmid constructs

The sequences of the *PRCC*, *NONO* and *TFE3* genes were obtained from GenBank. *PRCC‐TFE3* is fused by the first exon of *PRCC* and 4–10 exons of *TFE3*. *NONO‐TFE3* is fused by the first exons of exons 1–7 of *NONO* and exons 6–10 of *TFE3*. Then, the fusion genes were cloned into the pcDNA3.1‐3FLAG plasmid vector (Obio, H353). Then, the pcDNA3.1(+)‐3Flag‐P2A‐EGFP plasmid (Obio, H2713) was digested with EcoRI and BspEI, and after fused with the HIS sequence, the *PRCC‐TFE3* and *NONO‐TFE3* genes were introduced into the expression vector pFLAG‐CMV2 using EcoRI and BspEI sites. Full‐length human *SUMO1* was amplified by polymerase chain reaction (PCR) and introduced into the pcDNA3.1(+)‐3Flag‐T2A‐mCherry plasmid (Obio, H2714). All constructs were confirmed by sequencing. Mutations of *PRCC‐TFE3* or *NONO‐TFE3* at K330R or K460R were generated by using the Fast‐Mutagenesis Kit V2 (Vazyme, C214) following the instructions, and the primers used for K330R and K460R are shown in Supplementary Table S1. pEGFP‐C1‐AR and pCMV‐ His‐tagged ubiquitin (His‐Ub) plasmid was purchased from the MiaoLing plasmid sharing platform (P20284 and P4836).

### 2.4 Immunoprecipitation (IP)

After washing with ice‐cold phosphate buffered saline, two million cells were collected, and the IP assay was performed by using a Pierce Magnetic Co‐IP Kit (Thermo Fisher Scientific, 88804) according to the manufacturer's protocol. To identify the SUMO modification of target proteins, 20 mM N‐ethylmaleimide (NEM; Sigma–Aldrich, E3876) supplemented with 200 μM iodoacetamide (IAM) (Sigma–Aldrich, I6125) was added to the lysis buffer. To identify the ubiquitination of target proteins, the proteasome inhibitor MG132 was added 6 h before cell lysis. The soluble fractions were incubated with 4 μg of antibody and magnetic beads for 2 h at room temperature.

### 2.5 Western blotting

Total protein was extracted with pre‐cooled radio‐immunoprecipitation assay (RIPA) lysis buffer (1 × protease inhibitor cocktail, 1 × phosphatase inhibitor cocktail, 150 mM NaCl, 50 mM Tris‐HCl, pH 7.4, 0.1% sodium‐dodecyl‐sulphate (SDS), 1% NP) for 30 min. Then, 20 mM NEM and 200 μM IAM were added to the lysis buffer to observe the SUMOylated protein. The cell lysate was centrifuged at 13 400 g for 15 min, and then the supernatant was subjected to (SDS) sample buffer (50 mM Tris‐HCl, pH 6.8; 2% SDS; 5 mM EDTA; 10% glycerol; 80 mM dithiothreitol; 1 mg/ml bromophenol blue) by boiling for 10 min. Cell lysates were separated using SDS–polyacrylamide gel and then transferred to polyvinylidene fluoride (PVDF) membrane (Millipore, ISEQ10100) by standard procedures and then immunoblotted using the indicated antibodies (Supplementary Table S2). The protein concentration was examined by a BCA Protein Quantification Kit (Vazyme, Nanjing, China, E112) were used with tuamine protein concentration, and Image J software (NIH) was used to calculated grayscale values of bands.

### 2.6 Chromatin IP (ChIP)

Pierce Agarose ChIP Kit (Thermo Fisher Scientific, 26156) was used to perform ChIP assay following protocol. The DNA level was quantified by quantitative real‐time PCR (qPCR), and the special primers for ChIP are shown in Supplementary Table S3.

### 2.7 RNA isolation and real‐time quantitative PCR

Total RNA was isolated using TRIzol extraction reagent (Vazyme, R401) and was then reverse‐transcribed using a Hiscript II Reverse Transcriptase master mixing kit (Vazyme, R201) according to the manufacturer's instructions. All primers (Supplementary Table S4) were synthesised by Tsingke Biological Technology. PCR amplicons were quantified by SYBR Green (Vazyme, Q711) using an ABI ViiA 7 Q‐PCR System (Applied Biosystems). The relative abundance was analysed using the 2 –(ΔΔCt) method and normalised against 18S rRNA.

### 2.8 Luciferase reporter assay

The promoter of target genes was cloned into the PGL3‐Basic vector. HEK293T cells were cultured and then transfected with a target promoter plasmid (containing firefly luciferase), AR, PRCC‐TFE3 or NONO‐TFE3 plasmid using Lipofectamine 2000. The PRL‐TK plasmid (Promega, E2241; 100:1 ratio) was transfected as an internal control. After transfection for 48 h, the cells were lysed with a Dual‐Luciferase Reporter Assay Kit (Vazyme, DL101), and luciferase activity was detected with a GloMaxTM 96 Microplate Luminometer (Promega). The primers used for the luciferase reporter assay are provided in Supplementary Table S5.

### 2.9 Immunohistochemistry (IHC)

Paraffin‐embedded tissue specimens were sectioned into 4‐mm sections. Following routine deparaffinisation, rehydration and blocking, the sections were incubated in primary antibodies against proteins of interest. On the second day, the sections were incubated in horseradish‐conjugated rabbit secondary antibodies, followed by diaminobezidin 3 (DAB) and haematoxylin staining.

### 2.10 Immunofluorescence (IF)

Cells grown on glass‐bottom culture dishes were fixed, permeabilised and blocked sequentially. Then, the cells were incubated with the indicated primary antibodies overnight and with secondary antibodies for 1 h. Glass bottom was mounted with 4',6‐diamidino‐2‐phenylindole (DAPI) (Beyotime, P0131). Fluorescent images were examined and photographed on a confocal microscope (Olympus FV3000 Confocal Laser Scanning Microscope).

### 2.11 N**uclear and cytoplasmic extraction**

Nuclear and cytoplasmic fractions were extracted with NE‐PER Nuclear and Cytoplasmic Extraction Reagents (Thermo Fisher Scientific, 78835). In brief, cells were collected using trypsin‐EDTA and then centrifuged at 500 g for 5 min. Pre‐cold cytoplasmic protein extraction reagents I and II were added to the cell pellet, and the tube was centrifuged for 5 min at a speed of 16 000 g to obtain a cytoplasmic extract from the supernatant. The pellet containing nuclei was suspended in nuclear protein extraction reagent and centrifuged at 16 000 g for 10 min.

### 2.12 Flow cytometry analysis

Cells were washed and collected routinely for the follow‐up work. PI/RNase Staining Buffer (BD Pharmingen, 550825) and an Annexin V‐FITC/PI staining Kit (Vazyme, A211) were used following the manufacturer's protocols. The samples were detected by FlowJo software (v7.0) on a BD FACSCalibur flow cytometer (BD Biosciences).

### 2.13 Cell growth assays

Cell growth was assessed using EdU staining or cell counting kit 8 (CCK8) assays. EdU staining was performed using a BeyoClick EdU‐594 kit (Beyotime). Before EdU staining, UOK 109 and 786‐O cells were incubated with EdU for 3 h, and UOK120 cells were incubated with EdU for 6 h. The positive rate was calculated as the percentage of EdU‐stained cells among the total cell count in random fields. CCK8 assays were performed at 37°C for 2 h, and the absorbance was measured at 450 nm.

### 2.14 Cell migration and invasion assay

Cell migration ability was measured with a wound scratch experiment or transwell cell migration assay with an 8‐μm pore size (Corning Life Sciences). For the wound scratch experiment, wound closure was quantitated by ImageJ (NIH), and the wound healing percentage was calculated as the ratio between the wound area and initial scratch area. For the transwell cell migration assay, 5 × 10^4^ cells were seeded into the upper chambers. After 24 h, 4% paraformaldehyde and 1% crystal violet were used to fix and stain the cells that migrated. Cells were observed and counted with a microscope 50i (Nikon). For the cell invasion experiment, the upper chambers were precoated with Matrigel (Biosciences) and then subjected to the protocol used for the cell migration assay.

### 2.15 Plate colony formation assay

Five hundred cells were plated in 3.5‐cm dishes. After 7 days of culture, Giemsa stain solution (Solarbio, G1015) was used to stain the cell colonies, and ImageJ software was used to quantify and count the number of visible colonies.

### 2.16 Statistical analysis

Data in the tables are expressed as the mean ± SEM from at least three independent experiments. Student's *t*‐test, chi‐square test, Fisher's exact test or one‐way analysis of variance were used to calculate the statistical significance by SPSS 23.0 (SPSS Inc.). Statistical significance was defined as two‐sided *p*‐values less than .05. Survival data were obtained from electronic medical records. Progression‐free survival (PFS) was defined as the survival time without relapse or progression since the initiation of surgery. Overall survival (OS) refers to the time interval between surgery and death. Kaplan–Meier analysis was performed by log‐rank test. Differences were considered statistically significant when *p* < .05.

## 3 RESULTS

### 3.1 Androgen promotes the proliferation and migration of UOK120 cells in an AR‐dependent manner

**FIGURE 1:**
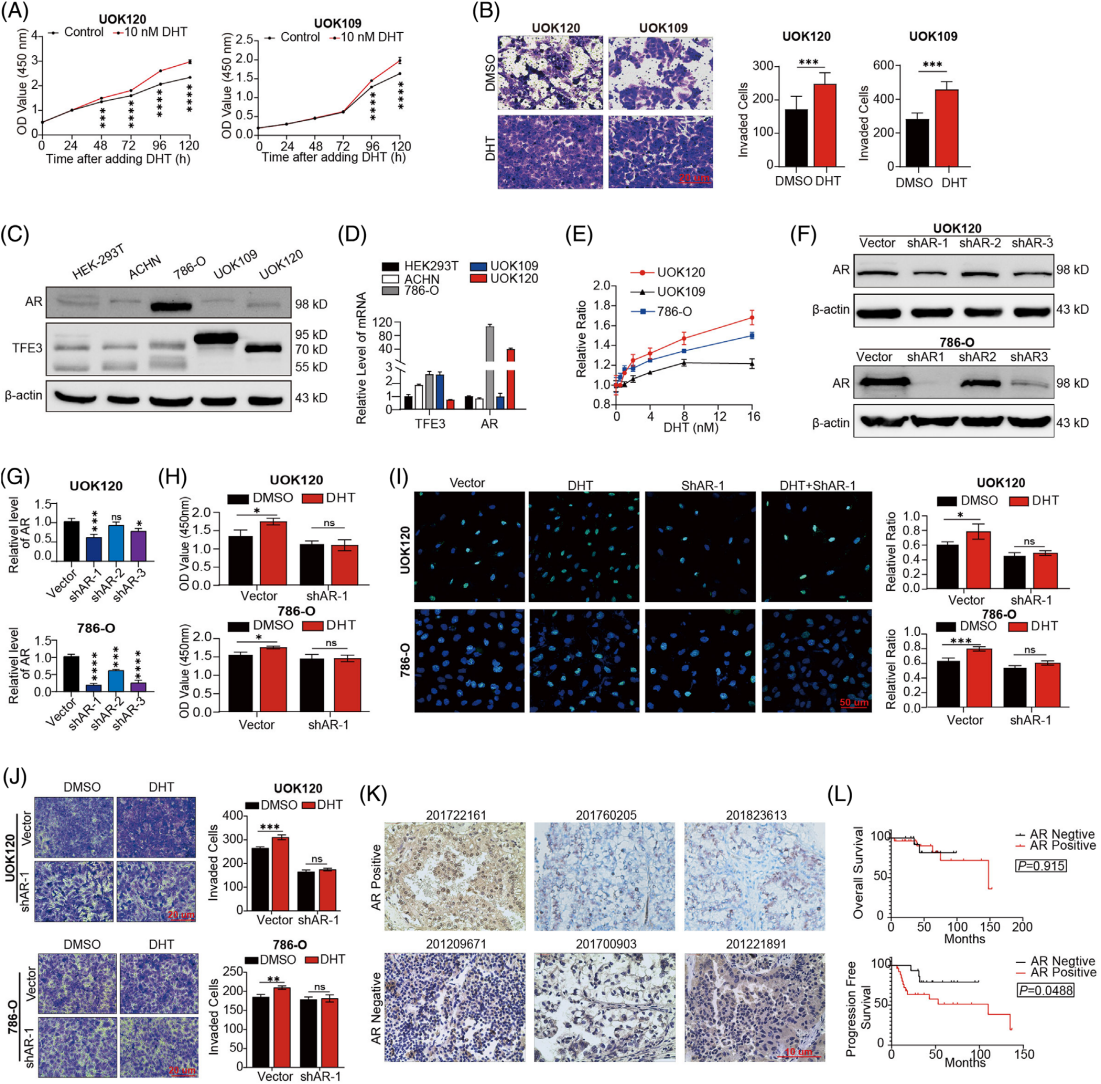
Effects of androgen and androgen receptor (AR) on Xp11.2 translocation renal cell carcinoma (Xp11.2 tRCC) and their effect on tumour proliferation and migration. (A) Cell proliferation abilities of UOK 120 and UOK109 cells were assayed by CCK‐8 after treatment with 10 nM DHT for 48 h. (B) Cell migration was determined by Transwell migration assay, and the average number of migrated cells was calculated. *N* = 3. (C) The expression of AR and TFE3 proteins in HEK‐293T, ACHN, 786‐O, UOK109 and UOK120 cells. (D) The transcriptional expression of *AR* and *TFE3* in HEK‐293T, ACHN, 786‐O, UOK109 and UOK120 cells. (E) 786‐O, UOK109 and UOK120 cells were treated with varying concentrations of DHT, and cell proliferation was assessed by CCK‐8 assay. Values were normalised against the OD value of the dimethyl sulfoxidetreated sample. F: UOK120 and 786‐O cells were transfected with three lentiviruses carrying shAR. (G) The efficiencies of three lentiviruses of shAR were quantified by the ImageJ software and normalised to the β‐actin protein level. (H) A CCK‐8 assay was performed to assess DHT cell proliferation after AR knockdown by lentiviral vectors or pLV‐shAR. *N* = 5. (I) EdU staining of UOK120 and 786‐O cells and EdU‐positive cell proportions are presented. Blue: DAPI; Green: EdU+; scale bar: 50 μm. *N* = 3. (J) Transwell migration assays and quantification of migrated cells. *N* = 3. (K) Representative immunohistochemistry (IHC) images of AR staining; scale bar: 20 μm. (L) Overall survival (OS) and progression‐free survival (PFS) of Xp11.2 tRCC patients according to AR staining. *N* = 46. ns: not significant. *p < .05, **p < .01, ***p < .001, ****p < .0001

The CCK‐8 assay showed that DHT promoted cell proliferation (Figure 1A). After treatment with DHT, the cell migration capacities of both UOK120 and UOK109 cells improved (Figure 1B) and apoptosis was reduced (Supplementary Figure S1A,B). In contrast, cell proliferation ability and the proportions of *S* phase cells decreased, and cell apoptosis increased after cells were cultured with CSM (Supplementary Figure S1C–G).

AR is the critical component of the androgen signalling axis. To compare the level of AR in Xp11.2 tRCC cells, we detected the relative expression of AR in several common renal cell lines. The obtained results showed that the protein and mRNA levels of AR in UOK120 and 786‐O cells were higher than those in HEK‐293T, ACHN and UOK109 cells (Figure 1C,D). Compared with UOK109 cells, UOK120 and 786‐O cells showed a more sensitive response to DHT (Figure 1E). When the expression of the AR gene was knocked down using three lentiviruses of shRNA, the most efficient lentivirus was selected (Figure 1F,G). The obtained results showed that the proliferative and promigratory effects of DHT were weakened after AR was knocked down (Figure 1H–J). Similar results were observed when UOK120 and 786‐O cells were treated with the AR inhibitors MDV3100 and ASC‐J9 (Supplementary Figure S2). Taken together, androgen promotes the proliferation and migration of UOK120 cells in an AR‐dependent manner. UOK120 cells showed decreased responses to MDV3100 after *TFE3* was knocked down (Supplementary Figure S2B), which further indicates the potential correlation between AR and TFE3 fusion proteins.

### 3.2 AR expression is negatively associated with PFS in Xp11.2 tRCC

To evaluate the predictive value of AR for the prognosis of Xp11.2 tRCC patients, the AR expression of 46 cases of Xp11.2 tRCC and 13 cases of ccRCC was detected by IHC, and the results showed that 26 (56.5%) cases of Xp11.2 tRCC and four (30.8%) cases of ccRCC were positive for AR (*p *= .101; Figure 1K). The association between the clinicopathological characteristics of Xp11.2 tRCC and AR expression is presented in Table 1. AR expression was negatively associated with PFS but not tumour size, tumour stage or pathological grade. Patients with AR expression presented worse PFS than patients without AR expression (*p *= .0488; Figure 1L). Based on the GEPIA online dataset (http://gepia.cancer‐pku.cn),^38^ we also analysed the expression of the AR gene in ccRCC, pRCC and ChRCC and found that the level of AR transcript expression in pRCC was significantly higher than that in normal regions (Supplementary Figure S3A). However, AR was negatively related to the tumour stage and was identified as a well‐described prognostic factor in conventional RCC types (Supplementary Figure S3B–D).

**TABLE 1:** The correlations between androgen receptor (AR) expression and clinicopathological factors

**Variables**	**No. of AR‐positive patients (26 cases)**	**No. of AR‐negative patients (20 cases)**	***p*‐value**
Age, year (mean ± SD)	35.3 ± 12.4	38.4 ± 14.4	.408
Gender			.655
Male	10	9	
Female	16	11	
Size, cm (mean ± SD)	5.0 ± 2.5	4.8 ± 2.0	.448
pT stage			.682
pT1‐2 stage	23	16	
pT3‐4 stage	3	4	
pN stage			.231
pN0	22	13	
pN1	4	7	
M stage			.955
M0	18	14	
M1	8	6	
AJCC stage			.202
I/II	19	11	
III/IV	7	9	
Fuhrman's grade			.266
1‐2 grade	12	6	
3‐4 grade	14	14	
Fusion partner			.385
ASPL	3	3	
PRCC	6	3	
SFPQ	3	2	
NONO	4	1	

### 3.3 Effects of AR on the expression level, intracellular localisation and transcriptional activity of TFE3 fusion proteins

**FIGURE 2:**
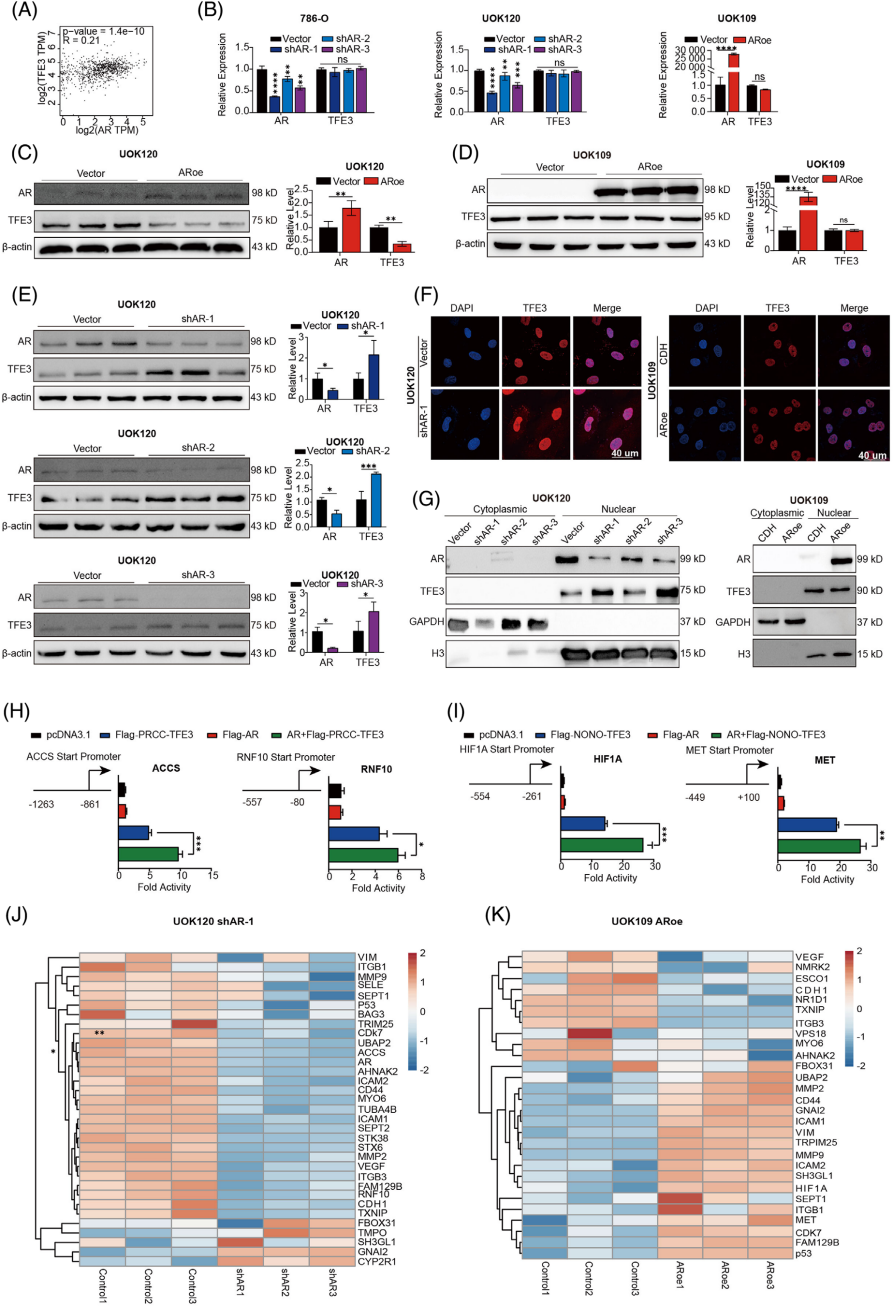
Effects of AR on the expression level, intracellular localisation and transcriptional activity of TFE3 fusion proteins. (A) Pairwise gene expression correlation analysis between *AR* and *TFE3* in RCC (including cleae cell RCC, pRCC and chrRCC) by GEPIA. (B) The relative transcriptional expression of *AR* and *TFE3*. (C–E): AR and TFE3 fusion proteins were detected by western blot, and β‐actin was used as a loading control. AR and TFE3 fusion protein levels were quantified by ImageJ software and normalised to β‐actin protein levels; (F) Cells were stained with TFE3 (red) followed by immunofluorescence photomicrographic analysis; scale bar: 40 μm. (G) Cytoplasmic and nuclear proteins were extracted and bolted. Glyceraldehyde‐3‐phosphate dehydrogenase (GAPDH) and histone H3 were used as loading controls for cytoplasmic and nuclear proteins, respectively. (H‐I) Reporter gene analysis using luciferase reporter constructs driven by the PRCC‐TFE3 (H) or NONO‐TFE3 (I) fusion protein binding sites; luciferase reporter activity was normalised to Renilla luciferase activity. (J‐K) The relative mRNA levels of direct target genes of TFE3 protein or TFE3 fusion proteins. shAR: AR knockdown with pLV‐shAR; ARoe: AR overexpression with pCDH‐AR. *N* = 3. ns: not significant. *p < .05, **p < .01, ***p < .001, ****p < .0001

As controlling the expression level, intracellular localisation and transcriptional activity of TFE3 fusion protein are critical steps for tumour progression of Xp11.2 tRCC, we first investigated the effect of AR on the expression level of wild‐type TFE3 and TFE3 fusions. The GEPIA database showed a weak correlation (OR = 0.21, *p *< .05) between AR and the wild‐type TFE3 gene (Figure 2A). Quantification results showed that the relative mRNA expression of *TFE3* in both Xp11.2 tRCC cells and non‐Xp11.2 tRCC cell lines did not significantly change if AR was overexpressed or knocked down (Figure 2B). However, western blot analysis showed that AR overexpression decreased the protein level of PRCC‐TFE3 but not NONO‐TFE3 or wild‐type TFE3 (Figure 2C,D, Supplementary Figure S4A,B). The level of the PRCC‐TFE3 fusion protein increased accordingly upon AR knockdown, unlike wild‐type TFE3 (Figure 2E, Supplementary Figure S4C,D). These results suggested that AR could alter PRCC‐TFE3 expression at the protein level instead of at the mRNA level (Figure 2B–E).

Next, we assessed whether AR could affect the intracellular localisation of wild‐type TFE3 and TFE3 fusions. IF microscopy and cytosolic/nuclear extract purification were performed to visualise the subcellular localisation of the TFE3 protein. The obtained results revealed that the subcellular localisation of TFE3 proteins was not affected by altered AR expression (Figure 2F,G, Supplementary Figure S4E,F).

As transcription factors, AR, PRCC‐TFE3 and NONO‐TFE3 fusion proteins modulate the transcription of their target genes by binding to E‐box sequences in regulatory regions. To assess the effect of AR on TFE3 fusion transcriptional activity, the ChIP‐Seq data of the three transcription factors were analysed to determine the same target genes (ChIP‐Seq data were obtained from PUBMED, PMID: 24981513 and 30849994). The obtained results showed that only a small number of genes were directly regulated by AR and TFE3 fusion proteins (Supplementary Figure S4G). The target genes *ACCS, RNF10, HIF1A* and *MET* were selected, and their reporter plasmids were constructed and cotransfected with plasmids encoding PRCC‐TFE3, NONO‐TFE3 or AR in HEK293T cells. The obtained results showed that the fluorescence intensity of the cotransfection groups was higher than that of the group transfected with AR or TFE3 fusions alone (Figure 2H,I, Supplementary Figure S4H). Using the database for annotation, visualisation and integrated discovery v6.8 (https://david.ncifcrf.gov/), the target genes of wild‐type TFE3 protein or TFE3 fusion proteins involved in the cell cycle and cell–cell adhesion were evaluated. qPCR results showed that with the overexpression or knockdown of AR expression, a large proportion of the target genes of the TFE3 fusion protein and wild‐type TFE3 correspondingly increased or decreased, respectively (Figure 2J,K, Supplementary Figure S4I). The change in target gene levels was consistent with the results of the luciferase reporter assay, showing a significant transcriptional regulatory role of AR on TFE3 fusions.

### 3.4 The TFE3 fusion protein can be SUMOylated in Xp11.2 tRCC

**FIGURE 3:**
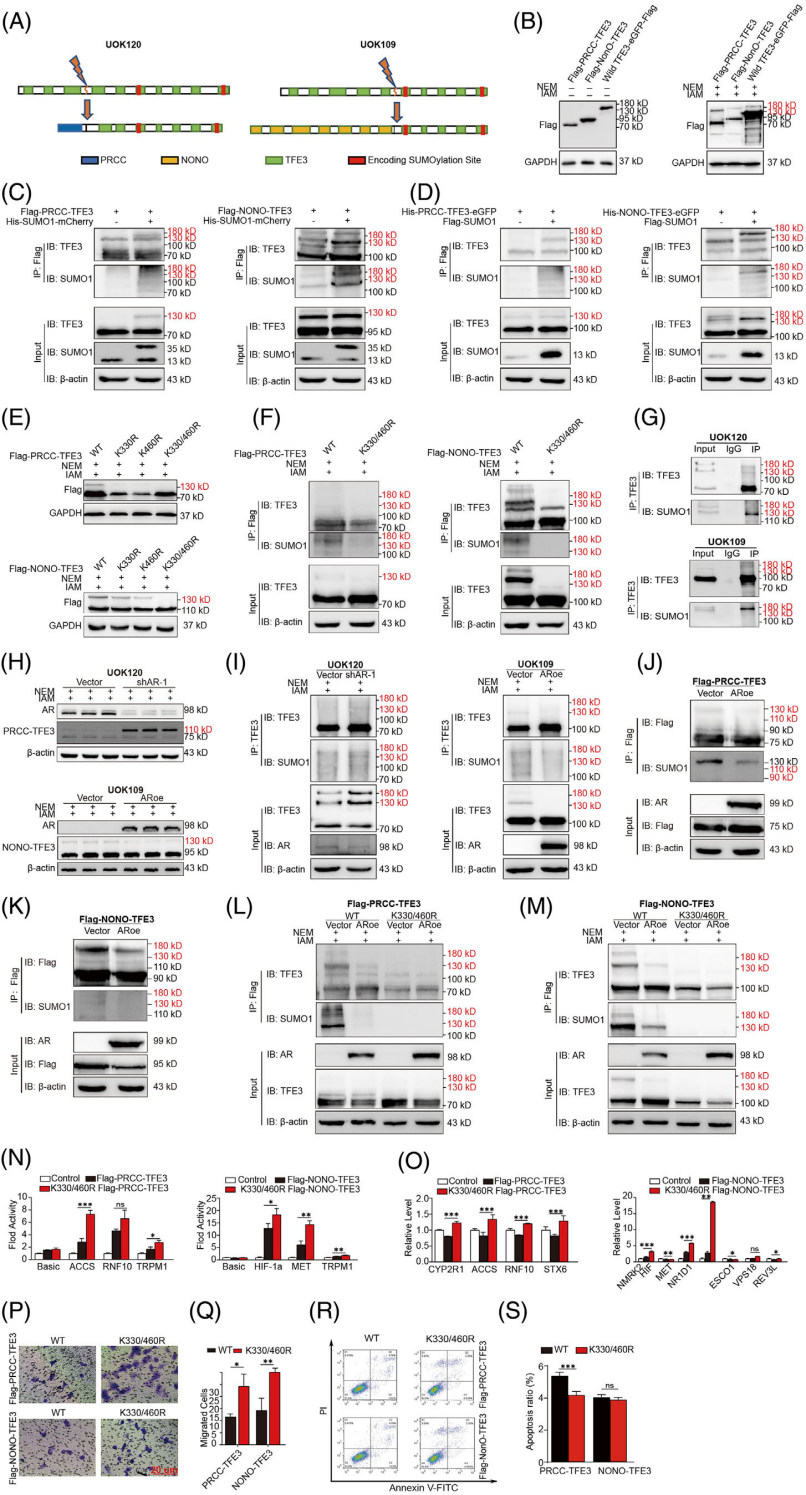
The influence of AR on the small ubiquitin‐related modifier (SUMO) modification of TFE3 fusion proteins. (A) Schematic representation of putative sites of SUMOylation in PRCC‐TFE3 (left) and NONO‐TFE3 (right) fusion proteins; (B) HEK293T cells were transfected with plasmids expressing Flag‐tagged PRCC‐TFE3, NONO‐TFE3 or wild‐type TFE3. Whole‐cell lysates with or without NEM and IAM were prepared for immunoblotting with anti‐Flag/anti‐GAPDH. (C‐D) HEK293T cells were transfected with plasmids expressing Flag‐tagged PRCC‐TFE3/NONO‐TFE3 together with His‐tagged SUMO1 or its empty vector control plasmid (C). In turn, plasmids expressing His‐tagged PRCC‐TFE3/NONO‐TFE3 together with Flag‐tagged SUMO1 or its empty vector control plasmid were also transfected into HEK293T cells (D). Forty‐eight hours after transfection, cell lysates with NEM and IAM were prepared and immunoprecipitated with anti‐Flag antibody. The immunoprecipitates were subjected to sodium‐dodecyl‐sulphate–PAGE and analysed by immunoblotting with anti‐TFE3 and anti‐SUMO1 antibodies. (E) Protein extracted from HEK293T cells cotransfected with plasmids encoding native‐type PRCC‐TFE3/NONO‐TFE3 or the indicated SUMOylated site mutants was extracted for immunoblotting with anti‐Flag antibody. (F) The SUMOylation of PRCC‐TFE3, NONO‐TFE3 or their mutants in HEK293T cells was assayed by immunoprecipitation (IP) with an anti‐Flag antibody. (G) SUMOylation of PRCC‐TFE3 or NONO‐TFE3 in UOK 120 cells and UOK 109 cells was assayed by IP with an anti‐TFE3 antibody. (H) Whole‐cell lysates of UOK120 and UOK109 cells stably expressing Flag‐AR or stably knocking down AR were obtained with NEM and IAM added. Upper bands with red markers indicate SUMOylated TFE3 fusion. (I) The SUMOylation of PRCC‐TFE3 and NONO‐TFE3 in UOK 120 cells and UOK 109 cells that stably expressed Flag‐AR or stably knocked down AR was assayed by IP with an anti‐TFE3 antibody. (J‐K): HEK293T cells with/without AR overexpression (ARoe/vector) were transfected with Flag‐PRCC‐TFE3 (J) or Flag‐NONO‐TFE3 (K), and after 48 h, whole cell lysates were prepared for immunoblotting with anti‐Flag. L‐M: HEK293T cells with/without AR overexpression (ARoe/vector) were transfected with Flag‐PRCC‐TFE3, Flag‐NONO‐TFE3 or their mutants, and after 48 h, whole cell lysates were prepared for immunoblotting with anti‐Flag. (N) Reporter gene analysis using a luciferase reporter construct driven by the PRCC‐TFE3 (left) or NONO‐TFE3 (right) fusion protein binding sites to assay the effect of SUMO modification on the transcriptional activity of TFE3 fusion proteins. Luciferase reporter activity was normalised to Renilla luciferase activity. (O) The relative mRNA level of direct target genes of TFE3 protein or TFE3 fusion proteins to assay the effect of SUMO modification on the transcriptional level of direct TFE3 target genes. (P‐Q): Migration assays in 786‐O cells transduced with plasmids encoding PRCC‐TFE3, NONO‐TFE3 or the indicated mutants. Quantification of migrated cells was compared by Student's *t*‐test. R‐S: The proportions of cells at the ‘S’ stage and the proportions of apoptotic cells are indicated. IAM, iodoacetamide; ARoe, AR overexpression with pCDH‐AR; NEM, N‐ethylmaleimide (NEM); shAR‐1, AR knockdown with pLV‐shAR‐1; WT, wild‐type NONO‐TFE3 or PRCC‐TFE3 fusion protein; K330/460R, NONO‐TFE3 or PRCC‐TFE3 fusion protein with K330/460R mutation. Upper bands with red markers indicate SUMOylated TFE3 fusion. *N* = 3. ns: not significant. *p < .05, ** *p* < .01, ***p < .001, ****p < .0001

Multiple studies have now established the association between AR and SUMOylation.^35^, ^36^, ^37^ We wondered whether TFE3 fusion proteins could be SUMOylated and whether AR moderated the transcriptional activity of PRCC‐TFE3 and NONO‐TFE3 by modifying SUMOylation. As the consensus amino acid sequence for SUMOylation is (I/L/V) KXE, we screened the sequence of the human wild‐type TFE3 protein and predicted two putative sites at lysine 330 and lysine 460. The genes encoding the two lysine residues are located in the sixth and 10th exons of *TFE3*. According to previous studies,^4^, ^39^, ^40^ the translocated *TFE3* fragments of TFE3 gene fusions in UOK109 and UOK120 cells contain exons from the sixth to the 10th exon (Figure 3A). After plasmids expressing Flag‐tagged PRCC‐TFE3, NONO‐TFE3 or wild‐type TFE3 were constructed and transfected into HEK293T cells, additional forms were observed at molecular masses of nearly 25 kD greater than the expected line in addition to the regular forms under the condition that the isopeptidase inhibitors NEM and IAM were added to the cell lysates (Figure 3B). Co‐IP showed that the slower migrating forms of PRCC‐TFE3 and NONO‐TFE3 were induced by their SUMOylation (Figure 3C,D). To further determine whether these lysine residues are still the major SUMOylation sites of the PRCC‐TFE3 and NONO‐TFE3 fusion proteins, Lys‐330 and Lys‐460 were conservatively replaced by arginine residues using site‐directed mutagenesis. The obtained results showed that after Lys‐330 and/or Lys‐460 were replaced, the upper bands of TFE3 fusion proteins decreased or disappeared (Figure 3E). IP results further identified that the SUMOylated form of TFE3 fusion proteins disappeared after both Lys‐330 and/or Lys‐460 were replaced (Figure 3F). These data suggested that K330 and K460 were functional SUMOylation sites for PRCC‐TFE3 and NONO‐TFE3 fusion proteins, respectively. In addition, IP showed that SUMOylation of TFE3 fusion proteins occurred in endogenous *PRCC‐TFE3* tRCC or *NONO‐TFE3* tRCC cells, UOK120 or UOK109 (Figure 3G). The SUMOylation ratio of NONO‐TFE3 in UOK109 cells was higher than that of PRCC‐TFE3 in UOK120 cells and wild‐type TFE3 in HEK293T, HK2 and 786‐O cells (Supplementary Figure S5A), and even the total expression levels of SUMO1 and SUMO2/3 were equivalent among 786‐O, UOK109 and UOK120 cells (Supplementary Figure S5B). Under cellular stress, such as heat shock, osmotic stress or oxidative stress, the SUMOylated ratio of PRCC‐TFE3 or NONO‐TFE3 clearly changed (Supplementary Figure S5C–F), which indicated that the SUMOylation of the TFE3 fusion protein occurred dynamically.

To clarify the influence of AR on the SUMOylation level of TFE3 fusion, NEM and IAM were added to cell lysates of UOK120 and UOK109 cells. Western blot analysis showed that AR upregulation/downregulation could alter the upper band of TFE3 fusion proteins in both UOK120 and UOK109 cells (Figure 3H). Co‐IP in both endogenous UOK120/UOK109 cells and exogenous HEK293T cells further identified a modification role of AR on the SUMOylation of PRCC‐TFE3 and NONO‐TFE3 fusion proteins (Figure 3I–K). Moreover, the modification role of AR on the K330/460R TFE3 fusion proteins disappeared (Figure L,M). In general, TFE3 fusion proteins could be SUMOylated, and AR moderated the transcriptional activity of TFE3 fusion proteins by modifying SUMOylation.

### 3.5 SUMOylation modulated the transcriptional activity of TFE3 fusions

Since SUMOylation is known to affect the function of transcription factors,^41^ additional experiments were performed to address whether SUMOylation influences the transcriptional activity of PRCC‐TFE3 and NONO‐TFE3. The reporter plasmids containing the promoter of target genes were cotransfected with plasmids encoding wild‐type or K330/460 mutant PRCC‐TFE3/NONO‐TFE3. The obtained results showed that K330/460R mutations were more capable of activating the reporter than wild types (Figure 3N). We also detected downstream target expression by qPCR and found a consistent result with the luciferase assay (Figure 3O). Then, the influence of SUMOylation on subcellular localisation and DNA blinding was also detected. The obtained results showed that deSUMOylation did not modulate the subcellular localisation of TFE3 fusions but improved the blinding levels of TFE3 fusions to their target genes (Supplementary Figure S6A–F). In addition, increased cell migration and colony‐forming capacity were observed in cells transfected with K330/460R types (Figure 3P,Q, Supplementary Figure S6G,H). Moreover, the cell apoptosis assay showed decreased cell apoptosis levels with deSUMOylated transfection, compared to native fusion proteins (Figure 3R,S). Collectively, the abovementioned data showed that the deSUMOylation of PRCC‐TFE3 or NONO‐TFE3 fusion promoted invasion by modulating their transcriptional activity, regardless of subcellular localisation and homodimerisation capacity.

### 3.6 AR regulates the SUMO modification of TFE3 fusion proteins by SENP1

**FIGURE 4:**
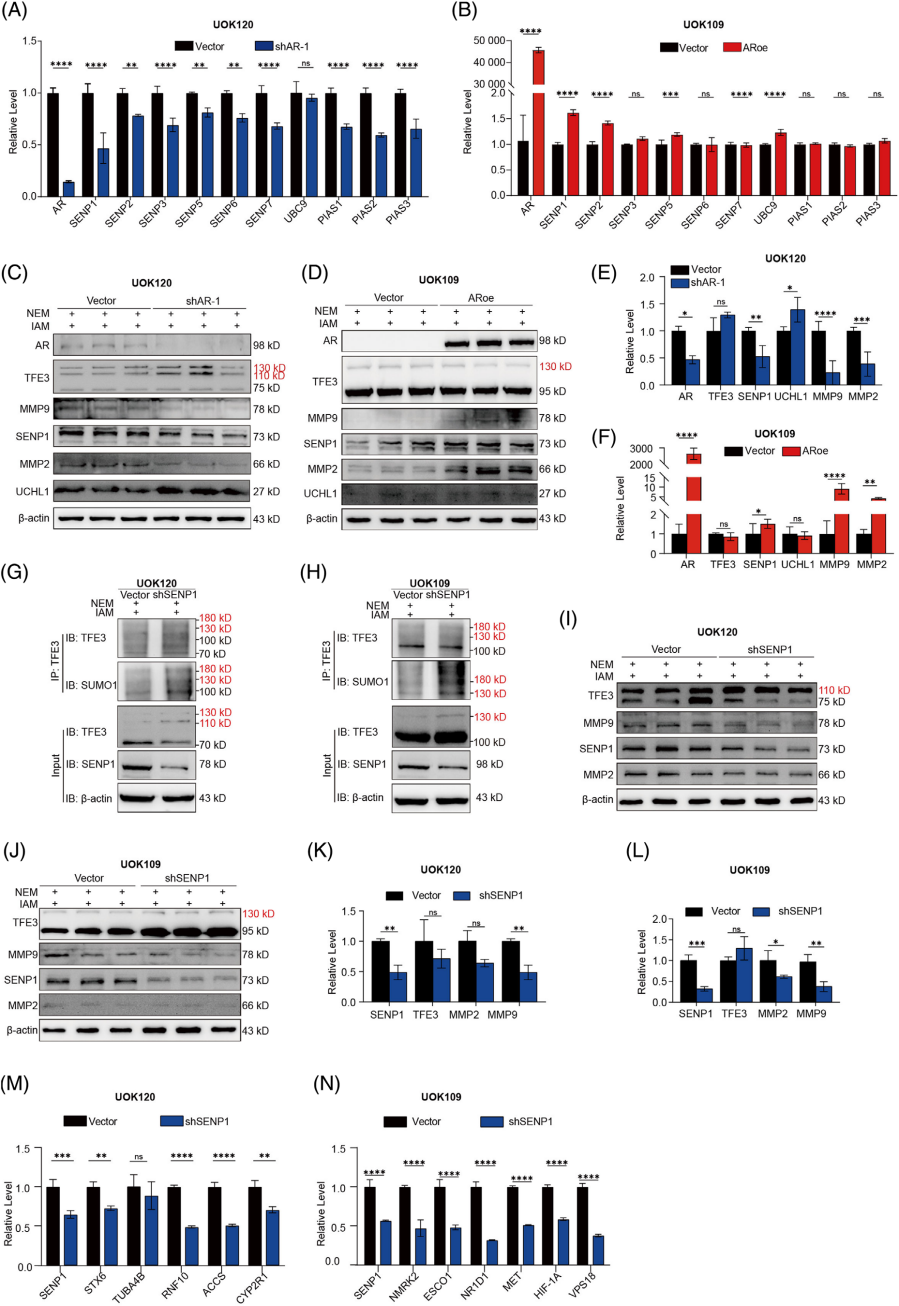
The role of SENP1 in the AR‐regulated SUMOylation of TFE3 fusion proteins. (A‐B) qPCR screened the enzymes involved in the process of SUMOylation and deSUMOylation upon changing the level of AR in UOK120 and UOK109 cells. (C‐F): Immunoblotting of SENP1, UCHL1, MMP2, MMP9 and TFE3 expression in UOK120 and UOK109 cells with AR knockdown or overexpression; β‐actin was used as a loading control. The relative levels of AR, TFE3, SENP1, UCHL1, MMP9 and MMP2 were quantified by ImageJ software and normalised to the β‐actin protein level. (G‐H The SUMOylation of PRCC‐TFE3 and NONO‐TFE3 in UOK 120 cells and UOK 109 cells with stable knockdown of AR was assayed by IP with an anti‐TFE3 antibody. (I–L) Immunoblotting of SENP1, MMP2, MMP9, SUMOylated and deSUMOylated TFE3 expression in UOK120 and UOK109 cells with SENP1 knockdown; β‐actin was used as a loading control. TFE3, SENP1, MMP9 and MMP2 were quantified by ImageJ software and normalised to the β‐actin protein level. (M‐N) The relative mRNA levels of target genes of PRCC‐TFE3 or NONO‐TFE3 in UOK120 and UOK109 cells with SENP1 knockdown. ARoe, AR overexpression with pCDH‐AR; NEM, N‐ethylmaleimide (NEM); shAR‐1, AR knockdown with pLV‐shAR‐1; shSENP1, SENP1 knockdown with pLV‐shSENP1; upper bands with red markers indicate SUMOylated TFE3 fusion. *N* = 3. Ns, not significant. *p < .05, **p < .01, ***p < .001, ****p < .0001

In mammalian cells, SUMOylation and deSUMOylation are reversible and highly dynamic processes by a series of enzymes. Target proteins are modified exclusively in a three‐step cascade mechanism, which requires the cooperation of enzymes E1, E2 and E3.^28^ Conversely, the isopeptide bond between SUMO and its target protein can be cleaved by SUMO‐specific proteases (SENPs) in a few seconds.^42^, ^43^, ^44^ We screened the enzymes involved in SUMOylation and deSUMOylation by qPCR and found that SENP1 changed the most with the knockdown or overexpression of AR (Figure 4A,B). Western blot analysis also confirmed the positive regulatory interactions between AR and SENP1 (Figure 4C–F, Supplementary Figure S7A,B). Furthermore, we identified that knockdown of *SENP1* improved the ratio of SUMOylated TFE3 fusions (Figure 4G,H) and decreased the MMP2/MMP2 level (Figure 4I–L) and mRNA level of the target genes of TFE3 fusions (Figure 4M,N). Our additional results showed that the overexpression of SENP1 reversed the SUMOylation inhibitory effect of AR on the PRCC‐TFE3 fusion and increased the levels of MMP2 and MMP9 (Supplementary Figure S7C,D).

### 3.7 AR facilitates degradation of the PRCC‐TFE3 fusion protein by affecting its ubiquitination

**FIGURE 5:**
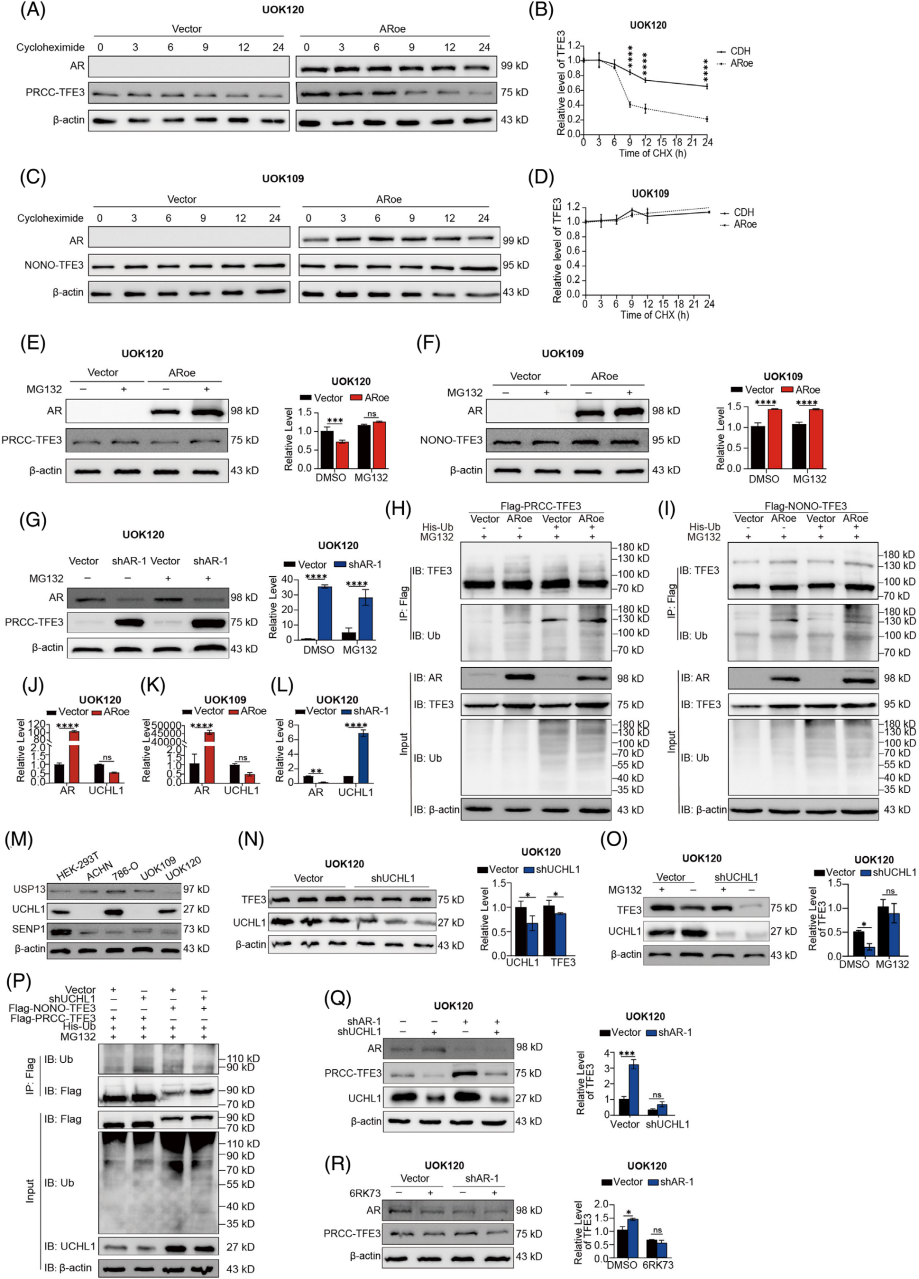
The influence of AR on the degradation of TFE3 fusion proteins. (A–D) UOK120 and UOK109 cells stably expressing Flag‐AR were treated with 60 mg/ml CHX. The cells were harvested at the indicated time points to measure TFE3 fusion protein levels. To quantify the TFE3 protein levels, actin was used for normalisation and then normalised to 0 h. Values represent three independent experiments. Statistical analyses were performed using two‐way ANOVA. (E–G) UOK120 and UOK109 cells with AR knockdown or expression were treated with 5 μM MG132 for 6 h before immunoblotting for TFE3 fusion. The relative level of TFE3 fusion was quantified and normalised to β‐actin. (H‐I) HEK293T cells with/without AR overexpression (ARoe/vector) were transfected with Flag‐PRCC‐TFE3 (H) or Flag‐NONO‐TFE3 (I), as well as His‐tagged ubiquitin (His‐Ub). MG132 (5 μM) was added 6 h prior to IP of TFE3 fusion proteins with a Flag antibody in both groups, followed by western blotting with a Flag and His antibody to examine TFE3 fusion ubiquitination. (J–L) qPCR screening of the relative level of UCHL1 upon changing the level of AR in UOK120 and UOK109 cells. (M) The expression of USP13, UCHL1 and SENP1 in HEK‐293T, ACHN, 786‐O, UOK109 and UOK120 cells. (N) UCHL1 in UOK120 cells was immunoblotted and quantified. β‐actin was used as a loading control. (O) UOK120 cells with UCHL1 knockdown were treated with 5 μM MG132 for 6 h before immunoblotting for TFE3 fusion. The relative level of TFE3 fusion was quantified and normalised to β‐actin. (P) HEK293T cells with/without UCHL1 knockdown (shUCHL1/vector) stability were transfected with Flag‐PRCC‐TFE3 or Flag‐NONO‐TFE3, as well as with His‐Ub. MG132 (5 μM) was added 6 h prior to IP of TFE3 fusion proteins with a Flag antibody in both groups, followed by western blotting with a Flag and Ub antibody to examine TFE3 fusion ubiquitination. (Q) UOK120 cells with/without UCHL1 knockdown (shUCHL1/vector) stability were then transfected with pLV‐shAR‐1/pLV‐Vec, and PRCC‐TFE3 was immunoblotted and quantified. β‐actin was used as a loading control. (R) UOK120 cells with/without UCHL1 knockdown (shUCHL1/vector) were treated with 6RK73 (5 μM), and PRCC‐TFE3 was immunoblotted and quantified. β‐actin was used as a loading control; ARoe, AR overexpression with pCDH‐AR; ANOVA, analysis of variance; CHX, cycloheximide; shAR‐1, AR knockdown with pLV‐shAR‐1. *N *= 3. Ns, not significant. *p < .05, **p < .01, ***p < .001, ****p < .0001

As the driver molecule of Xp11.2 tRCC, the level of TFE3 fusion proteins is closely related to the prognosis of Xp11.2 tRCC patients. The abovementioned results showed that AR suppressed the expression of the PRCC‐TFE3 fusion protein (Figure 2C–E). To elucidate the molecular mechanism, the stability of TFE3 fusion proteins was assessed, as the mRNA level of PRCC‐TFE3 seemed to not correlate with AR expression. To determine whether AR influenced the stability of the TFE3 fusion protein, UOK109 and UOK120 cells with stable knockdown or overexpression of AR were treated with cycloheximide to prevent de novo protein synthesis, and the total protein extract was extracted at the indicated time points. Indeed, overexpression or knockdown of AR significantly increased or decreased the degradation of the PRCC‐TFE3 fusion protein but not the NONO‐TFE3 fusion protein (*p* < .05; Figure 5A–D, Supplementary Figure S8A,B), which indicated that AR influenced the stability of the PRCC‐TFE3 protein. In addition, the effect of AR on PRCC‐TFE3 was eliminated in the presence of the proteasome inhibitor MG132 (Figure 5E–G). The obtained results revealed that AR could lead to the degradation of PRCC‐TFE3 in a proteasome‐dependent manner, indicating the role of AR in the ubiquitin‐mediated degradation of the PRCC‐TFE3 fusion protein. Then, we transfected Flag‐tagged PRCC‐TFE3 and His‐Ub into HEK293T cells that were stably infected with an AR‐overexpressing lentivirus or vector control in the presence of a proteasome inhibitor. IP assays indicated that ubiquitinated PRCC‐TFE3 accumulated in the cells with AR overexpression (Figure 5H). To our surprise, Flag‐tagged NONO‐TFE3 was transfected using a similar approach and showed slightly upregulated ubiquitination accumulation in the AR overexpression and control groups (Figure 5I). Given the phase separation^45^ and abnormal stability feature of NONO‐TFE3 (Figure 5C,D), we wondered whether the slight degradation of NONO‐TFE3 by AR might be masked and lead to contradictory results. Taken together, the degradation of the PRCC‐TFE3 fusion protein but not the NONO‐TFE3 fusion protein is affected by AR, although both PRCC‐TFE3 and NONO‐TFE3 can be ubiquitinated.

### 3.8 AR affects ubiquitination of the PRCC‐TFE3 fusion protein by negatively regulating UCHL1

A previous study found that AR increases MITF protein degradation by modulating ubiquitin‐specific protease 13 (USP13).^19^ Inconsistent with melanoma, the expression of AR showed no association with the level of USP13 (Supplementary Figure S8C,D). Then, we noted another study that found that MITF protein levels could be regulated through UCHL1.^46^ As a member of deubiquitinating enzymes, UCHL1 was initially discovered as a deubiquitinating enzyme while simultaneously bearing an E3 ubiquitin ligase under various conditions. Therefore, we examined UCHL1 expression upon changing the expression level of AR in UOK120 and UOK109 cells and found that AR negatively moderated the expression of UCHL1 in UOK120 cells, which provided a potential mechanism by which AR increased the ubiquitination of the PRCC‐TFE3 fusion protein by negatively regulating UCHL1 (Figures 4, 5 and Supplementary Figure S7A). Unlike UOK120 and 786‐O, the expression of UCHL1 was undetectable in UOK109 and ACHN cells by western blot (Figure 5M). Our additional results demonstrated that UCHL1 knockdown markedly deregulated the expression of the PRCC‐TFE3 fusion protein (Figure 5N). MG132 treatment noticeably weakened the effect of UCHL1 on the PRCC‐TFE3 fusion protein (Figure 5O). After Flag‐tagged PRCC‐TFE3 or NONO‐TFE3, as well as His‐Ub, were transfected into HEK‐293 cells that were stably infected with shUCHL1 lentivirus or vector control in the presence of MG132, the obtained results showed that ubiquitinated PRCC‐TFE3 accumulated more in the cells with UCHL1 knockdown than in the control cells (Figure 5P). Our additional results showed that UCHL1 knockdown or the specifically covalent irreversible UCHL1 inhibitor 6RK73 reversed the effect of AR on the PRCC‐TFE3 fusion protein (Figure 5Q–R). Together, the results in Figure 5 suggest that AR may suppress PRCC‐TFE3 fusion protein expression by increasing its degradation by negatively regulating UCHL1.

**FIGURE 6:**
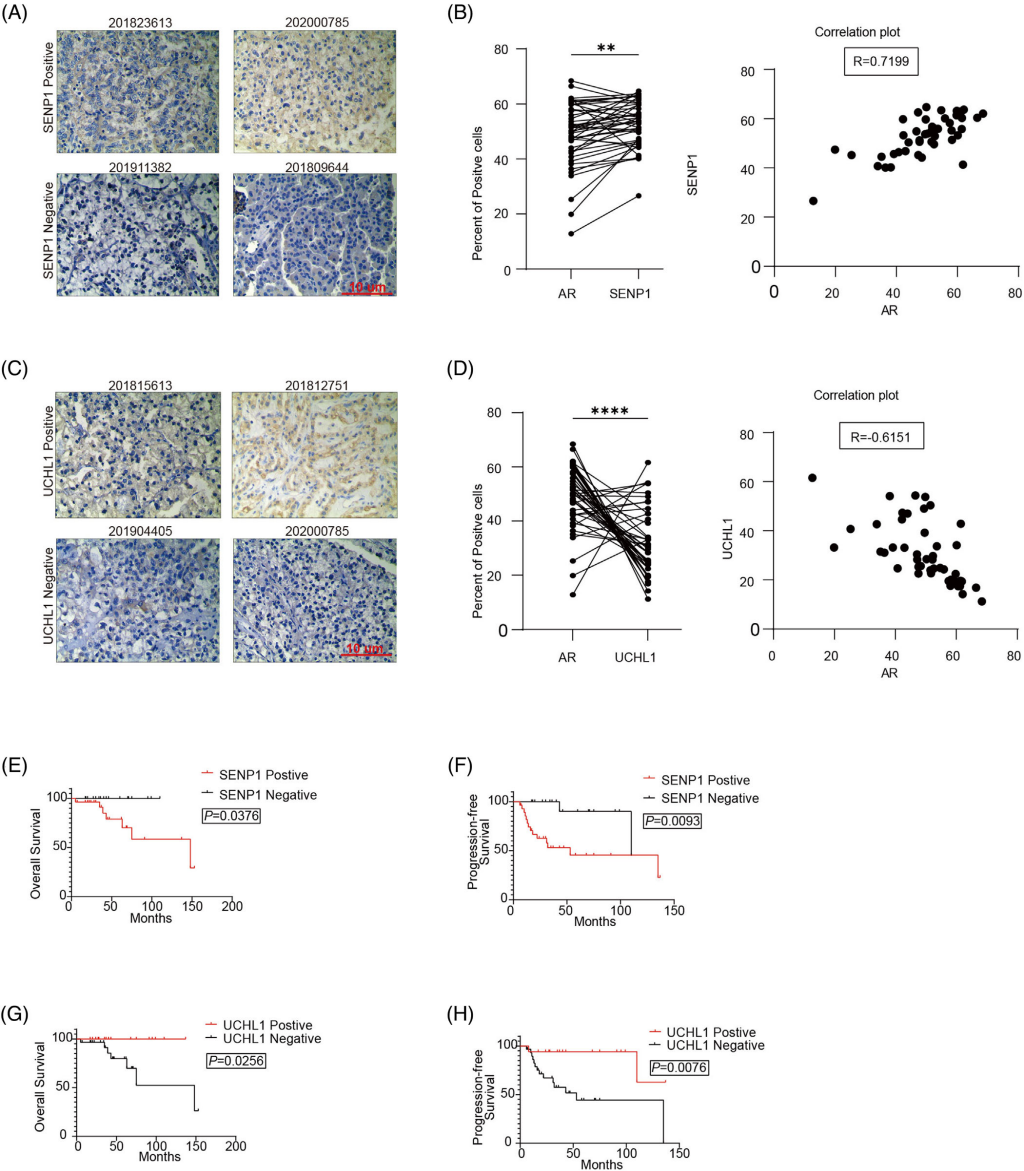
The association of UCHL1/SENP1 with AR and patient prognosis in Xp11.2 tRCCs. (A) Representative IHC images of SENP1 staining; scale bar: 10 μm. (B) Correlation between the expression levels of AR and SENP1 in Xp11.2 tRCCs. The positive proportion was quantified by ImageJ software. (C) Representative IHC images of UCHL1 staining; scale bar: 10 μm. (D) Correlation between the expression levels of AR and UCHL1 in Xp11.2 tRCCs. The positive proportion was quantified by ImageJ software. (E–H) OS and PFS of Xp11.2 tRCC patients according to SENP1 and UCHL1 staining

Then, IHC staining of SENP1 and UCHL1 was performed to evaluate their physiological relevance to AR and tumour progression (Figure 6A–D). Among 46 cases of Xp11.2 tRCC, 28 cases (60.9%) were positive for SENP1, and 17 cases (37.0%) were positive for UCHL1. In addition, the expression of AR was positively associated with SENP1 but negatively associated with UCHL1. The expression of UCHL1 was negatively associated with the AJCC stage (Table 2). The Kaplan–Meier survival analysis showed that SENP1 and UCHL1 were significantly associated with OS and PFS (Figure 6E–H). Among Xp11.2 tRCCs, SENP1 worked as a risk factor, and UCHL1 worked as a protective factor.

**TABLE 2:** The expression and relationship of UCHL1 and small ubiquitin‐related modifier‐specific proteases 1 (SENP1) with pathological stage, grade and prognosis in Xp11.2 translocation renal cell carcinoma tissues

**Variables**	**SENP1**			**UCHL1**		
	**Positive (28 cases)**	**Negative (18 cases)**	***p‐*value**	**Positive (17 cases)**	**Negative (29 cases)**	***p‐*value**
AR staining			.011			.026
Positive	20	6		6	20	
Negative	8	12		11	9	
AJCC stage			.152			.013
I/II	16	14		14	13	
III/IV	12	4		3	16	
Fuhrman's grade			.466			.399
1‐2 grade	8	7		8	10	
3‐4 grade	20	11		9	19	
Fusion partner			.571			.169
ASPL	3	2		3	3	
PRCC	2	4		2	7	
SFPQ	2	0		0	5	
NONO	1	3		1	4	

### 3.9 Targeting AR and UCHL1 delays tumour progression of Xp11.2 tRCC, alone or in combination

**FIGURE 7:**
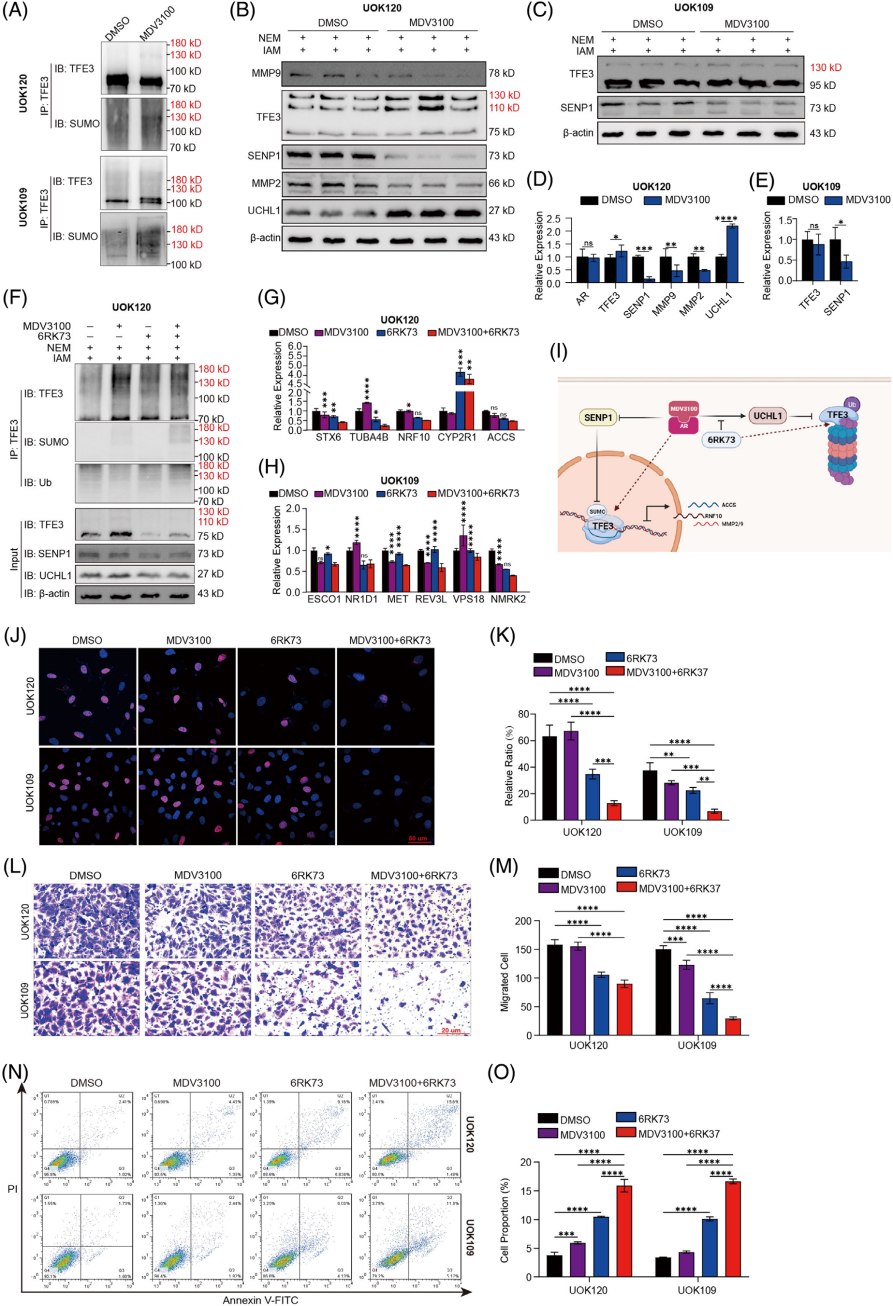
The effect of AR and UCHL1 inhibitors in Xp11.2 tRCC therapy. (A) After UOK120 and UOK109 cells were treated with MDV3100 (10 μM) for 48 h, the SUMOylation of PRCC‐TFE3 and NONO‐TFE3 was assayed by IP with an anti‐TFE3 antibody. (B–E) MDV3100 (10 μM) was added to UOK120 and UOK109 cells 48 h prior to immunoblotting. The relative levels of AR, TFE3, SENP1, MMP9, MMP2 and UCHL1 were quantified and normalised to β‐actin. (F) After UOK120 cells were treated with MDV3100 (10 μM) and/or 6RK73 (5 μM) for 48 h, the SUMOylation and ubiquitination of PRCC‐TFE3 were assayed by IP with an anti‐TFE3 antibody. (G‐H) The relative mRNA levels of target genes of PRCC‐TFE3 or NONO‐TFE3 in UOK120 and UOK109 cells after UOK120 and UOK109 cells were treated with MDV3100 (10 μM) and 6RK73 (5 μM) simultaneously for 48 h. The comparisons were single agent versus combined agents. (I) Graphical presentation of the proposed model for the regulation of transcriptional activity and degradation of TFE3 fusion by targeting AR and UCHL1. This figure was created with BioRender.com. (J‐K) EdU staining of UOK120 and 786‐O cells and EdU‐positive cell proportions are presented. Blue: DAPI; Red: EdU+; scale bar: 50 μm. (L‐M) Cell invasion was determined by Matrigel invasion assay with MDV3100 (10 μM) or 6RK73 (5 μM) added alone or in combination, and then the average number of migrated cells was calculated. (N‐O) Apoptotic cells were assessed by flow cytometry assay with MDV3100 (10 μM) or 6RK73 (5 μM) added alone or in combination. The percentage of apoptotic cells expresses the ratio of apoptotic cells to the total cell number. *N* = 3. Ns, not significant. *p < .05, ** *p* < .01, *** *p* < .001, ****p < .0001

To assess the therapeutic effects of AR inhibitors in suppressing the progression of Xp11.2 tRCC, UOK109 and UOK120 cells were treated with MDV3100, a second‐generation antiandrogen. The results showed that MDV3100 could improve the SUMOylation rate of TFE3 fusion proteins by reducing the expression of SENP1 (Figure 7A). At the same time, the level of total PRCC‐TFE3 fusion protein increased, followed by the improvement of UCHL1 (Figure 7B,D). However, the level of the NONO‐TFE3 fusion protein changed unremarkably owing to the lack of UCHL1 in UOK109 cells (Figure 7C,E). The combined use of MDV3100 and 6RK73 in UOK120 cells increased both the SUMOylation and ubiquitination of the PRCC‐TFE3 fusion protein (Figure 7F). Even though the SUMOylated ratio of the PRCC‐TFE3 fusion protein changed inconspicuously, the reduction in the mRNA level of PRCC‐TFE3 target genes was more pronounced in the combined group than in the individual agents (Figure 7G). The expression level of target genes in UOK109 cells treated with the combination of the drugs was not significantly changed compared with that in cells treated with MDV3100 or 6RK73 alone (Figure 7H). In addition, MDV3100 and 6RK73 had a significant effect on reducing the cell proliferation ability, cell invasion and migration capacity and promoting apoptosis of UOK 120 and UOK109 cells (Figure 7J–O). For UOK120 cells, the effect of combined medication on reducing cell invasion and migration capacity was better than monotherapy. Taken together, these results indicate a potential therapeutic modality for Xp11.2 tRCC by combinatorically targeting SUMOylation and ubiquitination (Figure 7I).

## 4 DISCUSSION

The crucial role of AR in the development and progression of prostate cancer has been acknowledged, and the expression of AR in susceptible male individuals, such as RCC and melanoma, has also been reported. However, the role of AR in the process of RCC remains controversial. As early as 2004, a study including 182 cases of RCC found that the positive rate of AR reached 14.8%, which was much higher than that of ERα (1.1%) and PR (1.1%). In addition, AR was regarded as a protective factor in RCC because the expression of AR was associated with lower tumour stage, unclear grade and prognosis. In contrast, a study in 2015 found that the mRNA expression level of AR was significantly higher in patients with pT2 tumours than in those with pT1 tumours. Treatment with DHT resulted in proliferation in the AR‐positive cell line HS891. T and CAKI2, while enzalutamide inhibited proliferation and cell viability in a dose‐dependent manner. A meta‐analysis including 11 retrospective studies with 1839 RCCs found no difference between AR expression and metastatic status, metastatic type (lymph or distant metastasis), susceptibility, pathological type or cancer‐specific survival of RCC. However, positive AR expression was demonstrated to be significantly associated with male patients, lower pathological grade and earlier tumour stage of RCC. In our study, relevant data extracted from GEPIA showed that AR expression was positively associated with PFS in conventional RCC, which indicated its tumour suppressor role in RCC. Our study discovered a relatively higher anti‐AR positivity ratio in Xp11.2 tRCC than in ccRCC (56.5% vs. 30.8%), although the difference was not statistically significant. However, AR expression was negatively associated with PFS in Xp11.2 tRCC patients but not the age of onset, patient sex, tumour size, tumour stage or pathological grade. In contrast, there was no significant difference in AR positivity between men and women, which could explain, at least in part, their semblable prognosis.

Recently, an in vitro study showed that AR increases haematogenous metastasis but decreases lymphatic metastasis of RCC by enhancing miR‐185‐5p expression, which promoted HIF2α/VEGF‐A and suppressed VEGF‐C expression.^47^ Therefore, anti‐AR combined with anti‐VEGF‐C compounds has the potential to be a better therapy for suppressing ccRCC progression. Another study discovered the increased expression of AR in RCC patients or RCC cell lines with either acquired or intrinsic tyrosine kinase inhibitor (RTKi) sunitinib resistance in vitro.^48^ Thus, targeting AR in combination with RTKi was an effective way to overcome the drug resistance of RCC. Regrettably, this study failed to reveal the association between AR expression and haematogenous metastasis or lymphatic metastasis in Xp11.2 tRCC tissues. Due to a lack of available comprehensive internal medicine data on Xp11.2 tRCC, it was unwarranted to attribute the insensitivity of RTKi to AR expression. However, our results first identified AR as a risk factor for Xp11.2 tRCC and provided a novel molecular mechanism for the age‐dependent prognostic difference in tumour progression. Previous studies on AR mainly concentrated on male susceptible tumours such as liver,^49^, ^50^ bladder^51^, ^52^ and kidney,^53^, ^54^ while Xp11.2 tRCC preferred to implicate females, especially those of reproductive age.^55^ Therefore, the effect of estrogen/progestogen and their receptors on the progression of Xp11.2 tRCC deserves more in‐depth investigation.

SUMOylation and deSUMOylation are reversible and highly dynamic processes that are influenced by cellular stress.^42^, ^43^ To avoid the release of SUMO from target proteins by endogenous isopeptidases, the combination of the isopeptidase inhibitors NEM and IAM in the current study was traditionally employed to study the SUMOylation rate of TFE3 proteins once preparing cell lysates.^31^, ^32^, ^56^ A series of studies proved that MiT/TFE could be SUMOylated by SUMO1 in melanocytes and renal cells, which led to attenuated melanocyte and renal cell clonogenicity.^31^, ^32^ In this study, a series of in vitro experiments were conducted and identified that TFE3 fusions, including PRCC‐TFE3 and NONO‐TFE3, maintained the SUMOylation site. After mutating both SUMOylation sites in the TFE3 fragment, the SUMOylation level of TFE3 fusions was completely abolished. Furthermore, it was confirmed that the transcriptional activity of TFE3 fusion proteins increased due to deSUMOylation.

SENP1 was reported to play an important role in regulating AR‐dependent transcription and hypoxia signalling by removing SUMO isoforms from AR, and the expression of SENP1 directly correlates with the aggressiveness and recurrence of prostate cancer by regulating two critical bone remodelling proteins, MMP2 and MMP9.^35^ In turn, the transcription of *SENP1* is significantly elevated with chronic androgen exposure in the androgen‐sensitive human prostate cancer cell line, indicating the feedback mechanism between SENP1 and AR,^36^ which might explain the overexpression of SENP1 both in prostate cancer tissue samples and precancerous prostate intraepithelial neoplasia lesions.^37^ Similar to prostate cancer, our study found that both AR and SENP1 were highly expressed in Xp11.2 tRCC, and the transcript activity of the TFE3 fusion could be regulated by SENP1. Although our study identified PIAS3, an E3 ligase that was reported to interact with MITF and repress its transcriptional activity,^32^, ^57^, ^58^ which could also be regulated by AR, SENP1 was the most notable one after knocking down or overexpressing AR in Xp11.2 tRCC cell lines. Treatment with the AR antagonist enzalutamide was proven to be an effective therapeutic intervention for Xp11.2 tRCC by promoting the SUMOylation of TFE3 fusions. Thus, our data provide insight into the link between SUMOylation of TFE3 fusion proteins and Xp11.2 tRCC.

To date, only three ubiquitin enzymes have been reported to regulate MiT/TFE degradation.^46^, ^59^, ^60^ STUB1, a chaperone‐dependent E3 ubiquitin ligase, was reported to target phosphorylated TFEB under conditions of mTOR inhibition^59^; however, our previous study identified that both PRCC‐TFE3 and NONO‐TFE3 escaped phosphorylation regulation by mTOR, and most TFE3 fusions maintained the phosphorylation site.^22^ As a deubiquitination enzyme, USP13 was reported to stabilise and upregulate MITF protein levels by altering its deubiquitination.^60^ Further in vitro and in vivo studies found that AR increased MITF protein degradation by modulating miRNA‐539‐3p/USP13 signalling, which resulted in increased melanoma cell invasion by increasing the expression of the receptor tyrosine kinase AXL.^19^ Inconsistent with melanoma, the data presented herein failed to demonstrate the role of AR in regulating USP13. UCHL1, with ubiquitin hydrolase and ligase activity, is another reported enzyme participating in controlling MITF stability.^46^, ^61^ In a study of human melanocytes, UCHL1 was reported to inhibit the expression of MITF by working as an E3 ligase.^46^ In contrast, this study identified a function of the ubiquitin hydrolase UCHL1 in reducing the degradation of the PRCC‐TFE3 fusion protein in UOK120 cells. In general, the ubiquitination‐promoting activity of UCHL1 is mainly shown in the context of neurodegenerative disorders, such as α‐synuclein degradation in Parkinson's disease^61^ or amyloid β‐precursor protein accumulation in Alzheimer's disease.^62^ In vitro studies suggested that the ubiquitin ligase function of UCHL1 was dimerisation‐dependent and ubiquitin concentration‐dependent, and ligase activity was inhibited when serine 18 was mutated to tyrosine (S18Y).^61^, ^63^ However, histidine 161 was the catalytic site of UCHL1 when it worked as a ubiquitin hydrolase.^64^ In this way, the catalytic site and ubiquitin concentration might be responsible for the opposing models of UCHL1 in melanocytes and Xp11.2 tRCC. Our in vitro experience further identified that the UCHL1 inhibitor 6RK73 alone not only facilitated the degradation of the TFE3 fusion protein in UOK120 cells but also eliminated the accumulation of PRCC‐TFE3 after treatment with MDV3100.

## 5 CONCLUSION

Overall, this study identified that AR was frequently expressed in Xp11.2 tRCC tissues and that the expression of AR was associated with patient PFS. AR induced transcriptional activity of TFE3 fusion proteins and degradation by regulating their deSUMOylation and ubiquitination, respectively. Furthermore, UCHL1 inhibitor 6RK73‐induced degradation of the PRCC‐TFE3 fusion protein led to enhancement of the potency of the AR antagonist enzalutamide in UOK120 cells. These findings provide a rationale for the clinical testing of combination strategies with AR and UCHL1 inhibitors in Xp11.2 tRCC.

## CONFLICT OF INTEREST

The authors declare that they have no competing interests.

## Supporting information



Supporting Information

Supporting Information

Supporting Information

Supporting Information

Supporting Information

Supporting Information

Supporting Information

Supporting Information

Supporting Information

Supporting Information

Supporting Information

Supporting Information

Supporting Information
