# Supplementary figures and images for: Both SUMOylation and ubiquitination of TFE3 fusion protein regulated by androgen receptor are the potential target in the therapy of Xp11.2 translocation renal cell carcinoma
Source: Clin Transl Med. 2022 Apr 22;12(4):e797. doi: 10.1002/ctm2.797 (PMC9029019; doi:10.1002/ctm2.797)

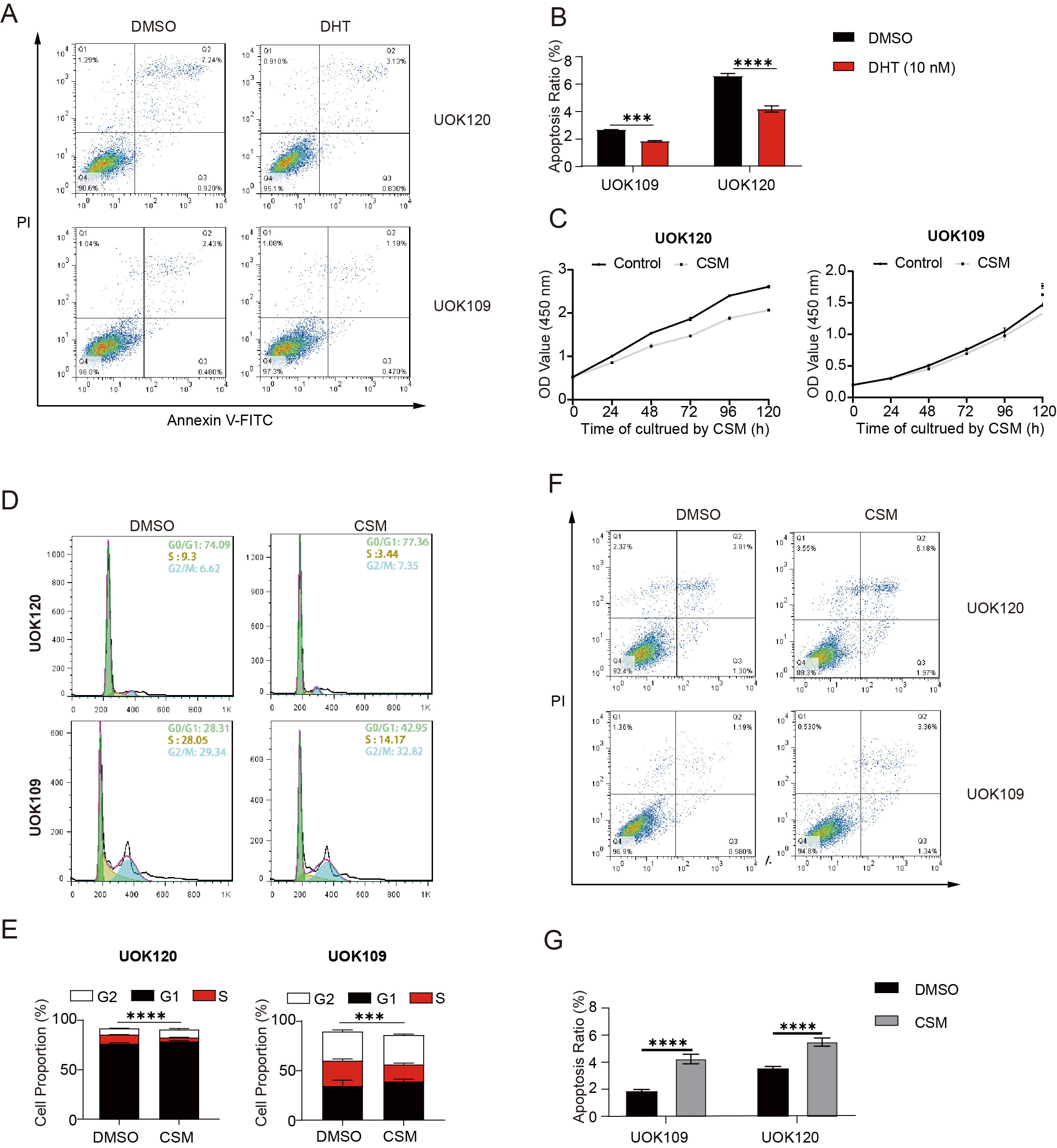

Supplement: Supplementary file 1 — Supporting Information [file CTM2-12-e797-s012.jpg]

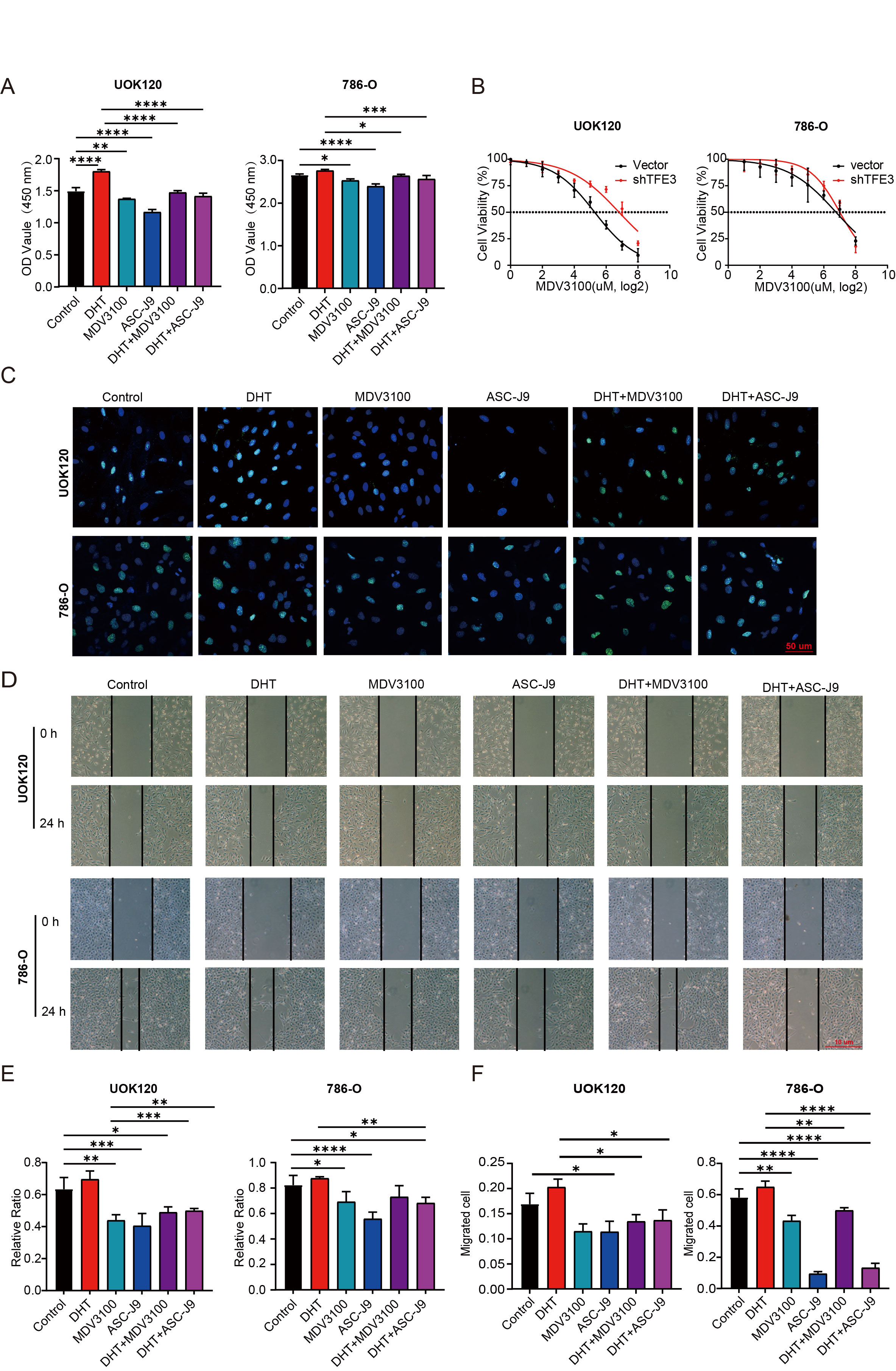

Supplement: Supplementary file 2 — Supporting Information [file CTM2-12-e797-s002.jpg]

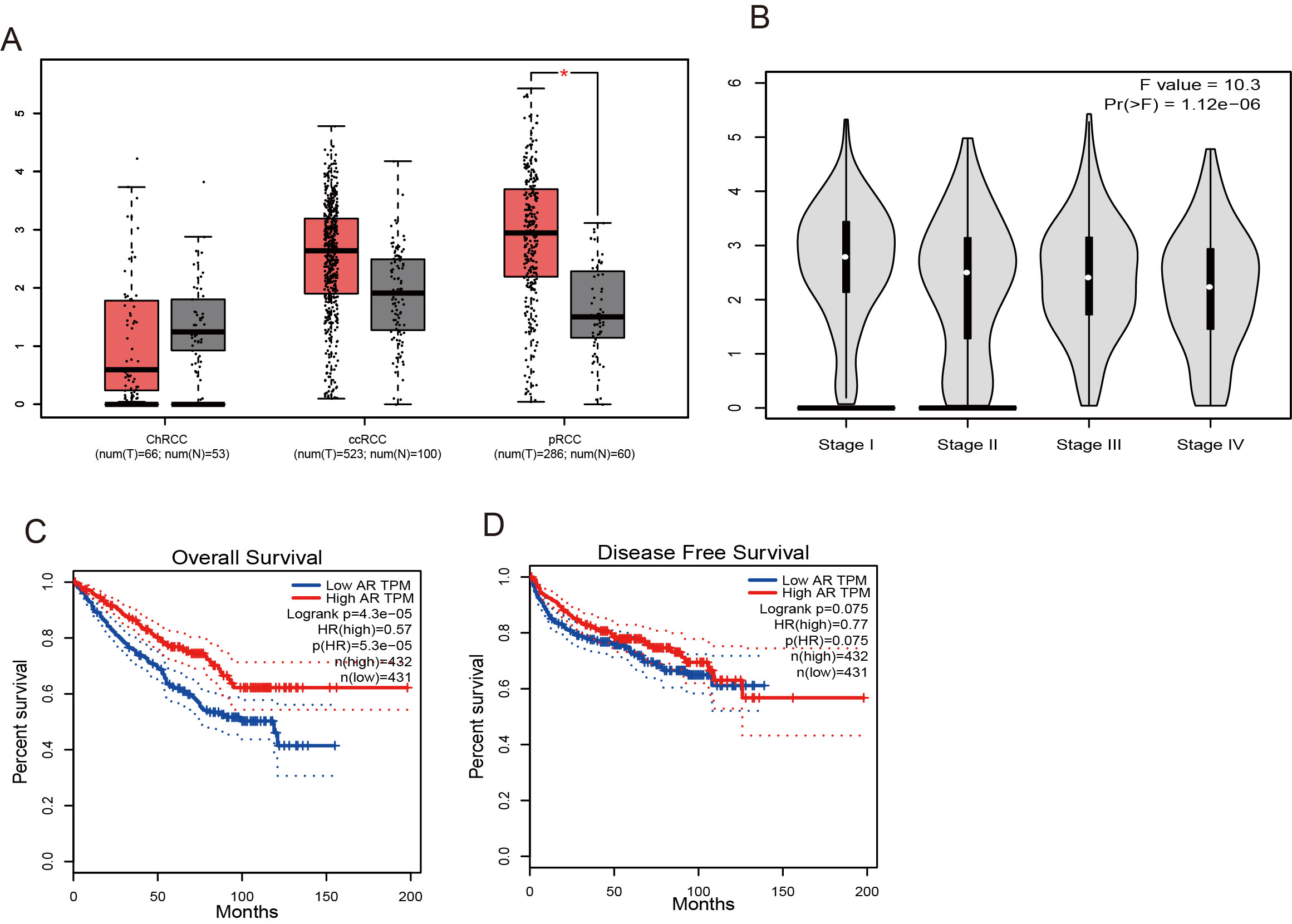

Supplement: Supplementary file 3 — Supporting Information [file CTM2-12-e797-s013.jpg]

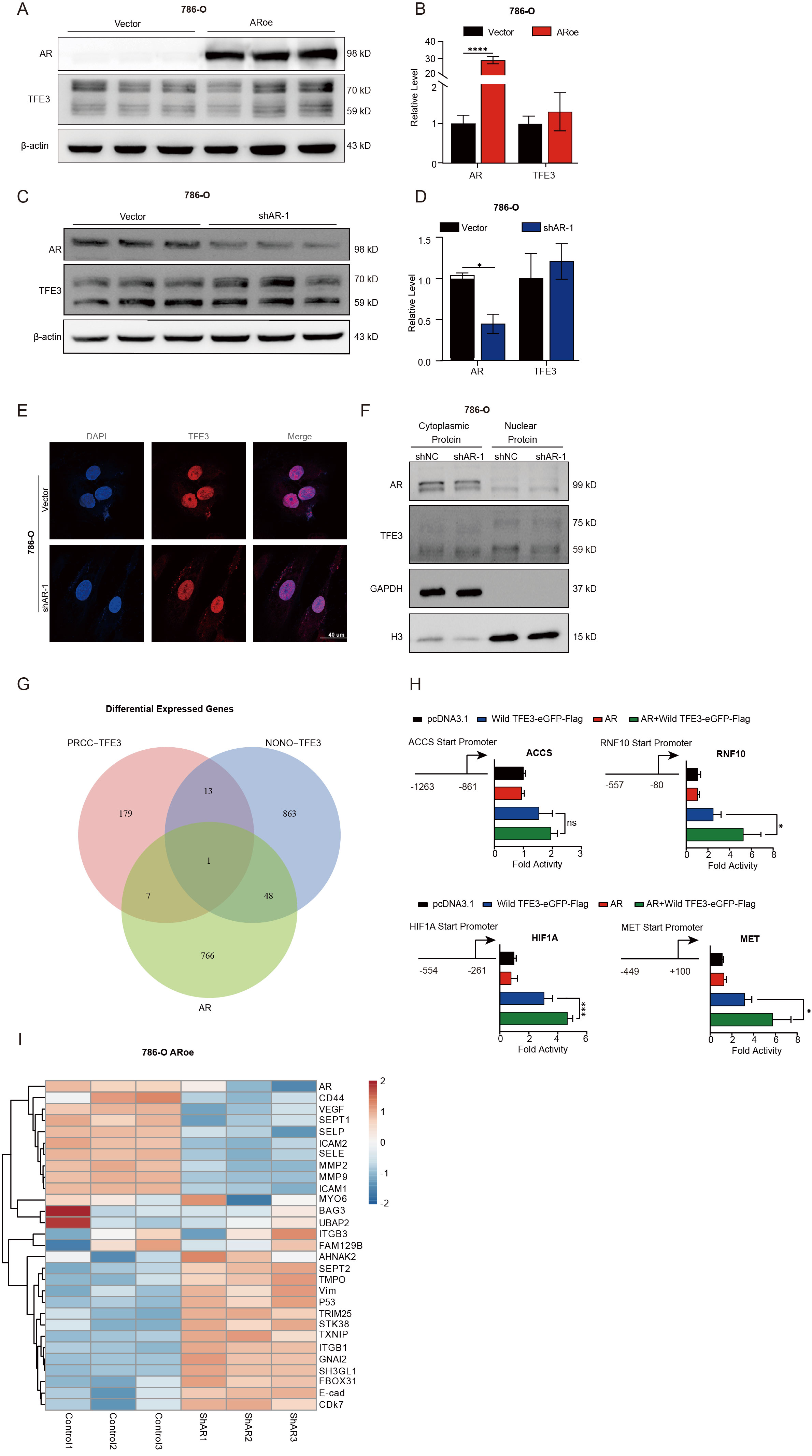

Supplement: Supplementary file 4 — Supporting Information [file CTM2-12-e797-s011.jpg]

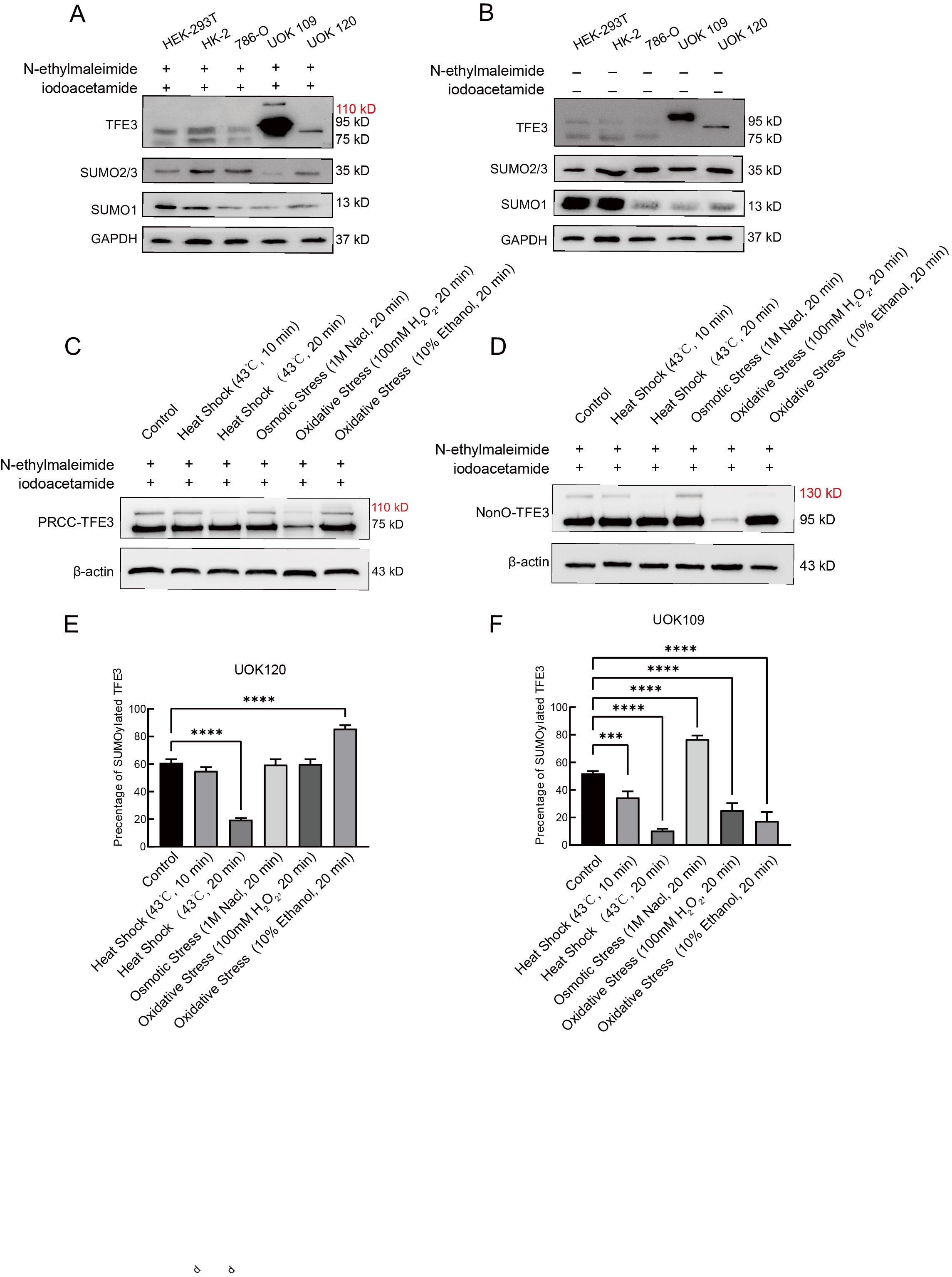

Supplement: Supplementary file 5 — Supporting Information [file CTM2-12-e797-s004.jpg]

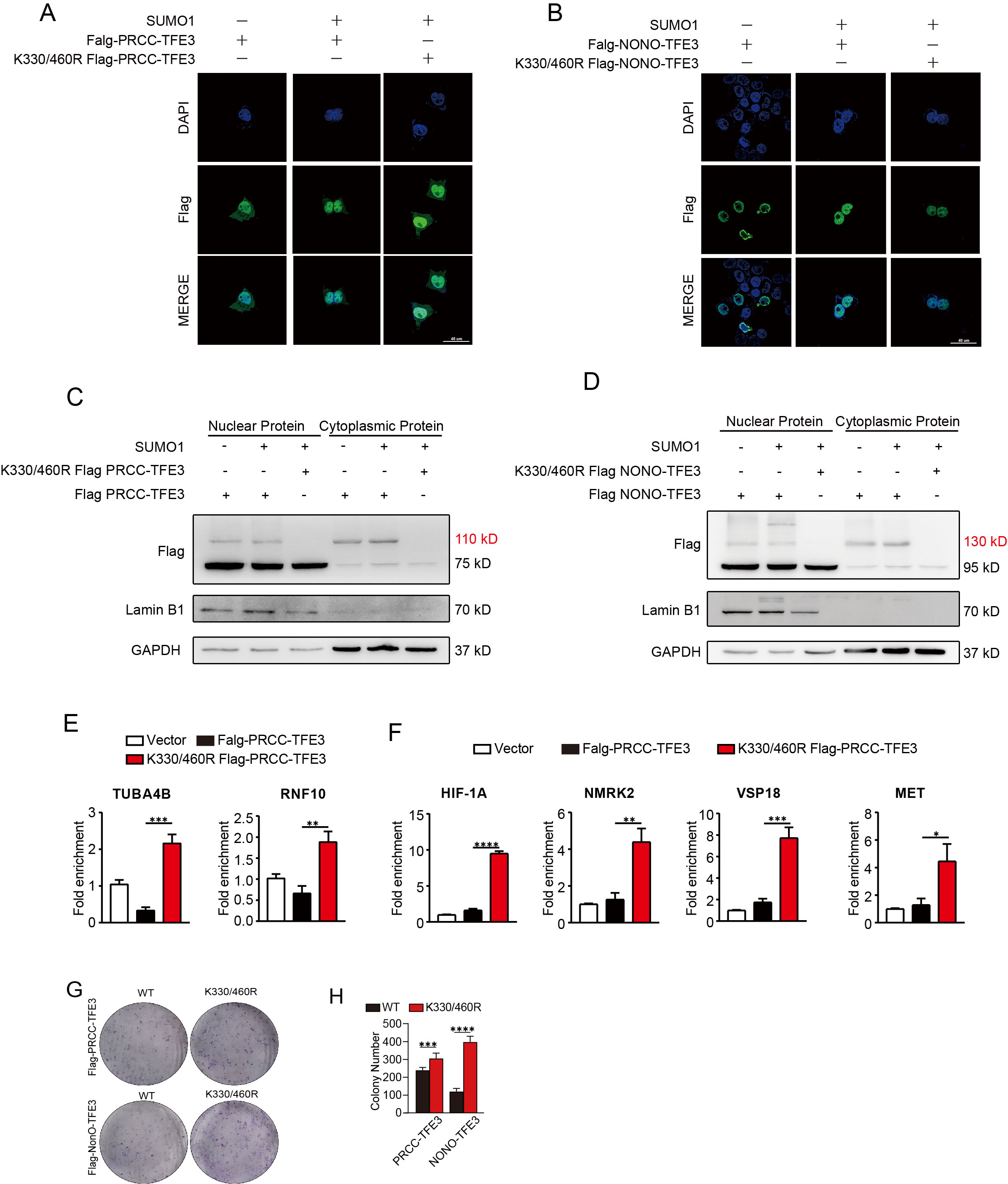

Supplement: Supplementary file 6 — Supporting Information [file CTM2-12-e797-s003.jpg]

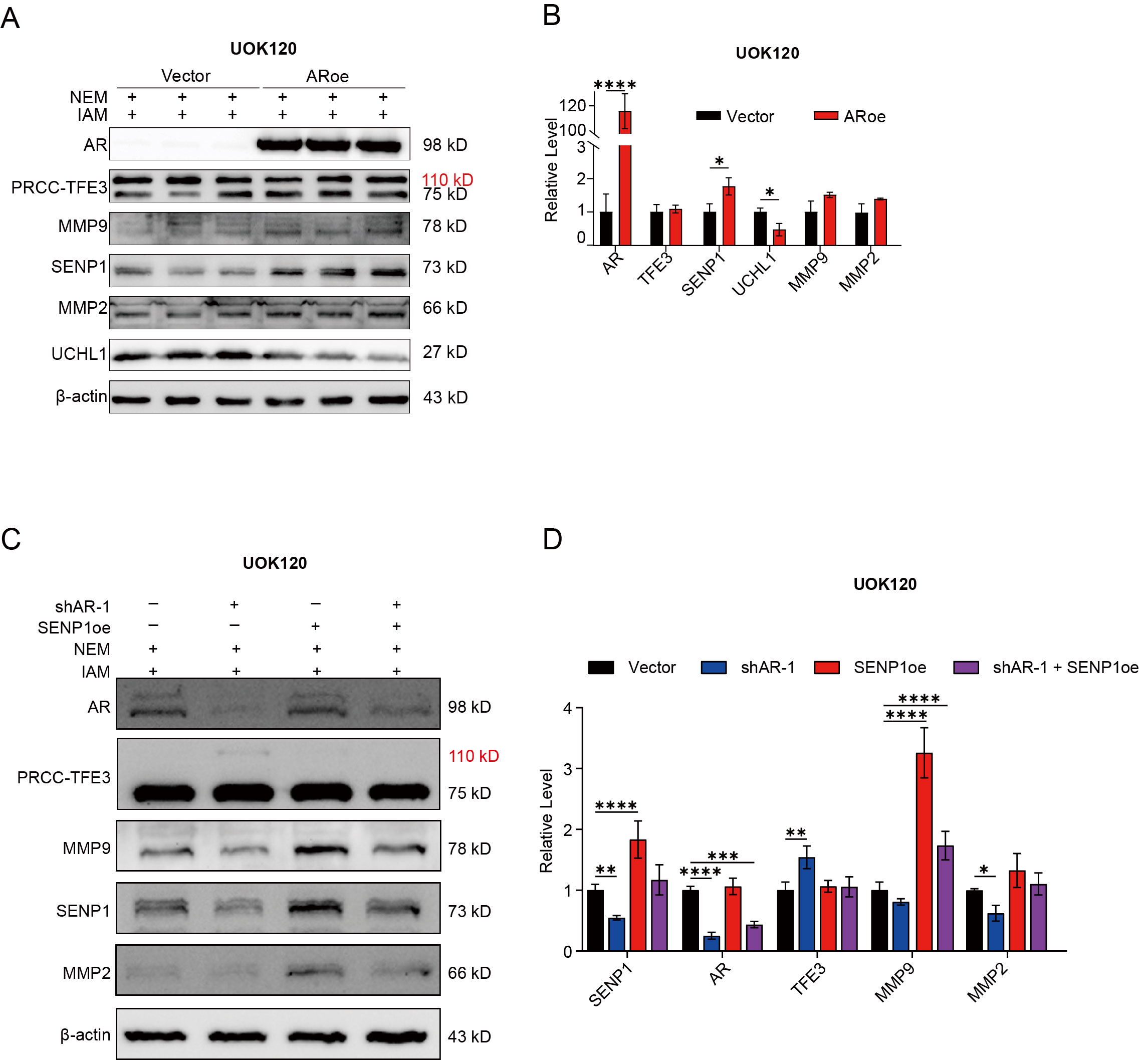

Supplement: Supplementary file 7 — Supporting Information [file CTM2-12-e797-s009.jpg]

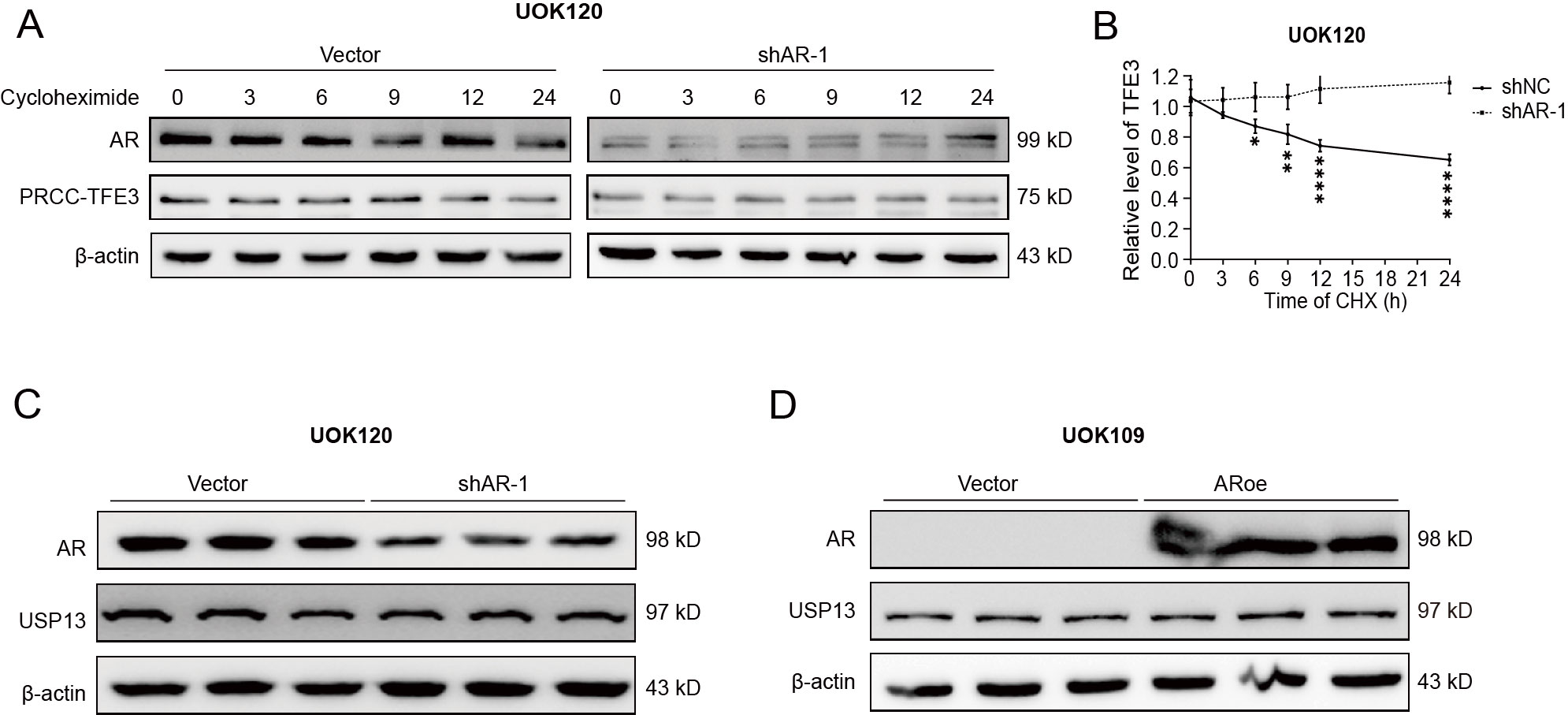

Supplement: Supplementary file 8 — Supporting Information [file CTM2-12-e797-s007.jpg]
