# Supplementary material for: Both SUMOylation and ubiquitination of TFE3 fusion protein regulated by androgen receptor are the potential target in the therapy of Xp11.2 translocation renal cell carcinoma
Source: Clin Transl Med. 2022 Apr 22;12(4):e797. doi: 10.1002/ctm2.797 (PMC9029019; doi:10.1002/ctm2.797)
Supplement: Supplementary file 9 — Supporting Information [file CTM2-12-e797-s005.docx]

**Supplement Table 1.** Primers used for SUMOylation sites mutations.

| Target  promoter | Primer sequence (5’-3’) | |
| --- | --- | --- |
|  | Forward | Reverse |
| K330R | CAACATCAgACGGGAGATCTCTGAGACCGAGG | TCTCCCGTcTGATGTTGGGCAGCTCAGCTGGG |
| K460R | TTCTGACAGCCTCAgGCCAGAGCAGCTGGACATTG | GCcTGAGGCTGTCAGAAGCCGAAGTCGTGGCC |
