# Supplementary material for: Both SUMOylation and ubiquitination of TFE3 fusion protein regulated by androgen receptor are the potential target in the therapy of Xp11.2 translocation renal cell carcinoma
Source: Clin Transl Med. 2022 Apr 22;12(4):e797. doi: 10.1002/ctm2.797 (PMC9029019; doi:10.1002/ctm2.797)
Supplement: Supplementary file 10 — Supporting Information [file CTM2-12-e797-s006.docx]

**Supplement Table 2.** The information of antibodies.

| Immunogen | Sourse | Company | Catalog number |
| --- | --- | --- | --- |
| GAPDH | Mouse | Proteintech, Wuhan, China | 60004-1-Ig |
| VEGF | Mouse | Santa Cruz Biotechnology | sc-7269 |
| MMP9 | Mouse | Santa Cruz Biotechnology | sc-21733 |
| SUMO1 | Mouse | Santa Cruz Biotechnology | sc-5308 |
| SUMO2/3 | Mouse | Santa Cruz Biotechnology | sc-393144 |
| Ubiquitin | Mouse | Abcam | ab7254 |
| β-actin | Rabbit | ABclonal, Wuhan, China, | AC026 |
| UCHL1 | Rabbit | ABclonal, Wuhan, China, | A20380 |
| Flag | Rabbit | Proteintech, Wuhan, China | 20543-1-AP |
| DYKDDDDK affinity gel | Rabbit | YEASEN, Shanghai, China | 20585ES03 |
| E cadherin | Rabbit | Proteintech, Wuhan, China | 20874-1-AP |
| Histone H3 | Rabbit | affinity | BF9211 |
| MMP2 | Rabbit | Proteintech, Wuhan, China | 10373-2-AP |
| His | Rabbit | Proteintech, Wuhan, China | 66005-1-Ig |
| SENP1 | Rabbit | Proteintech, Wuhan, China | 25349-1-AP |
| USP13 | Rabbit | Proteintech, Wuhan, China | 16840-1-AP |
| GFP | Rabbit | Cell Signaling Technology | 2956 |
| Lamin B1 | Rabbit | Cell Signaling Technology | 13435 |
| TFE3 | Rabbit | Abcam | ab93808 |
| TFE3 | Rabbit | Sigma | HPA023881 |
| AR | Rabbit | Abcam | ab209491 |
| HRP-conjugated goat anti-rabbit |  | Cell Signaling Technology | 7074 |
| goat anti-mouse secondary antibody |  | Boster | BA1050 |
| Alexa Fluor 488-conjugated goat anti-rabbit secondary antibodies |  | Abcam | ab150077 |
| Alexa Fluor 594-conjugated goat anti- rabbit secondary antibodies |  | Invitrogen | A-11058 |
