# Supplementary material for: Both SUMOylation and ubiquitination of TFE3 fusion protein regulated by androgen receptor are the potential target in the therapy of Xp11.2 translocation renal cell carcinoma
Source: Clin Transl Med. 2022 Apr 22;12(4):e797. doi: 10.1002/ctm2.797 (PMC9029019; doi:10.1002/ctm2.797)
Supplement: Supplementary file 11 — Supporting Information [file CTM2-12-e797-s010.docx]

**Supplement Table 3.** Primers used for ChIP assay.

| Gene | Primer | Sequence（5’-3’） |
| --- | --- | --- |
| TUBA4B | Forward | AGTCCTCACCTTGCGAGTCT |
|  | Reverse | TCTAGCTGTAAACCCATACCTCT |
| RNF10 | Forward | AACCTGGACTTGGAGCAACTG |
|  | Reverse | TGGAGAGGGGGTGAGGTATT |
| HIF-1A | Forward | CATGTTTGGGACCAGGCAAC |
|  | Reverse | GCTCTCAGCCAATCAGGAGG |
| MET | Forward | AGTTTCACCTTGTCGTGGGC |
|  | Reverse | CCAGGCGACCAGACTGAG |
| NMRK2 | Forward | AGGCATTTGTAGCCCCTGTG |
|  | Reverse | CCCAGTTTCGGTGGCAAGAA |
| VPS18 | Forward | GGCTGGTGAGAGTCACAGTT |
|  | Reverse | CTTAGGCGACCCAGATGGAG |
