# Supplementary material for: Both SUMOylation and ubiquitination of TFE3 fusion protein regulated by androgen receptor are the potential target in the therapy of Xp11.2 translocation renal cell carcinoma
Source: Clin Transl Med. 2022 Apr 22;12(4):e797. doi: 10.1002/ctm2.797 (PMC9029019; doi:10.1002/ctm2.797)
Supplement: Supplementary file 12 — Supporting Information [file CTM2-12-e797-s001.docx]

**Supplement Table 4.** Primers used for real-time PCR.

| Gene | Primer | Sequence（5’-3’） |
| --- | --- | --- |
| 18s RNA | Forward | TTGCCCTCCAATGGATCCT |
|  | Reverse | GTGTTTGTCAGGTTCAACTCCA |
| TFE3 | Forward | AGTGCCCAGTTCCTTGATCC |
|  | Reverse | GTACAAGAGCATGATCCTGGA |
| AR | Forward | CCAGGGACCATGTTTTGCC |
|  | Reverse | CGAAGACGACAAGATGGACAA |
| CYP2R1 | Forward | CTGCCATTTATCGGCAACATCT |
|  | Reverse | GGAAGGCATGGTCTGTCTGC |
| ACCS | Forward | GAAGGAGAATGCTCCAGAAAACT |
|  | Reverse | CCATGTGGTAGGTCCTGTAGC |
| RNF10 | Forward | CGGCGAGTCTAAACCCAAGAG |
|  | Reverse | AGGAAAGTTCACGTTTGCGATTA |
| STX6 | Forward | CACCAACGAGCTGAGAAATAACC |
|  | Reverse | CCCTGACAACTTGCCGAGT |
| HIF-1A | Forward | GAACGTCGAAAAGAAAAGTCTCG |
|  | Reverse | CCTTATCAAGATGCGAACTCACA |
| MET | Forward | AGCAATGGGGAGTGTAAAGAGG |
|  | Reverse | CCCAGTCTTGTACTCAGCAAC |
| NR1D1 | Forward | TGGACTCCAACAACAACACAG |
|  | Reverse | GATGGTGGGAAGTAGGTGGG |
| NMRK2 | Forward | ACTGCTGCGTGATCCATCAG |
|  | Reverse | TCCCACTGTTTGAAGCCGTC |
| ESCO1 | Forward | AGAATTGGAAACACGCATGAGT |
|  | Reverse | GATCTCCGGTTAAGCTGTTCATT |
| VPS18 | Forward | CACTCGGGGTATGTGAATGCC |
|  | Reverse | TCGGAAGGGGTGAAGTCAATG |
| REV3L | Forward | AATGTGGCTTTAGGCAATCCA |
|  | Reverse | AGGTAGGGAATATGCGCTTCA |
| STUBA4B | Forward | GATGTATGCCAAGAGGGCGT |
|  | Reverse | CCCCCACTCCACACTATCCA |
| SENP1 | Forward | AGTGAACCACAACTCCGTATTC |
|  | Reverse | AAAAGATCGGTCCAAATGTCCTT |
| SENP2 | Forward | GGCTGGTTAGGATTCTCGGC |
|  | Reverse | GGCAGCATTGTAGAGACTGTTTT |
| SENP3 | Forward | CCCGACCCTCTTTTGATGCC |
|  | Reverse | GCAGGTTTTTCGATGAGTGGG |
| SENP5 | Forward | GGGAAGGCCAGTTACTTGGAA |
|  | Reverse | CAAAGGGGTTCATCCTTGATCC |
| SENP6 | Forward | TCCTGTAAGGTTAAGTCGGCT |
|  | Reverse | AGATAGAGGAGGAGTAGGCTGAT |
| SENP7 | Forward | GGCCATCTTCATCCGAAATCA |
|  | Reverse | CAAAGGGAGAGTCCAGCGT |
| UBC9 | Forward | AAAAATCCCGATGGCACGATG |
|  | Reverse | CTTCCCACGGAGTCCCTTTC |
| PIAS1 | Forward | ACAGTGCGGAACTAAAGCAAA |
|  | Reverse | GGACTTGAATGTACGTTGGGG |
| PIAS2 | Forward | ATCCACGAACTCTTGAAGGACT |
|  | Reverse | TGTGGGCTTAGTATCTTGAAGCA |
| PIAS3 | Forward | CTGGGCGAATTAAAGCACATGG |
|  | Reverse | AAAGCGTCGTCGGTAAAGCTC |
| SEPT1 | Forward | GGAGTCATGGACAAGGAGTACG |
|  | Reverse | ACCCCTTCTTGACAGACTTGC |
| FBOX31 | Forward | GCCGTGAGGAGTATGGTGTTT |
|  | Reverse | GTACATCCACCCGATGATGAAC |
| GANI2 | Forward | TACCGGGCGGTTGTCTACA |
|  | Reverse | GGGTCGGCAAAGTCGATCTG |
| TXNIP | Forward | GGTCTTTAACGACCCTGAAAAGG |
|  | Reverse | ACACGAGTAACTTCACACACCT |
| CDK7 | Forward | ATGGCTCTGGACGTGAAGTCT |
|  | Reverse | GCGACAATTTGGTTGGTGTTC |
| SH3GL1 | Forward | GGGCAAGATCCCCGATGAG |
|  | Reverse | CACCTGCTCGATGTCAGTCTC |
| MYO6 | Forward | TATTGTGGATATTGGCCCCGA |
|  | Reverse | TGGATTCACTGCAATCAGAATGT |
| TRPIM25 | Forward | AATCGGCTGCGGGAATTTTTC |
|  | Reverse | TCTCACATCATCCAGTGCTCT |
| FAM129B | Forward | CTGACGGAGTTCCTCCAGTTC |
|  | Reverse | GAGGTTCCCCGAGAAGACGAT |
| SEPT2 | Forward | CTAAGCAACAGCCAACTCAGT |
|  | Reverse | TTTCGGTGAACTTGATTGGGG |
| TMPO | Forward | CCCCTCGGTCCTGACAAAAG |
|  | Reverse | CGCTCTTCGTCACTGGAGAA |
| STK38 | Forward | ACAAAGGAAAGGGTGACAATGAC |
|  | Reverse | CTTCCGAGCATGTGCTGATCT |
| BAG3 | Forward | TGGGAGATCAAGATCGACCC |
|  | Reverse | GGGCCATTGGCAGAGGATG |
| UBAP2 | Forward | GCAGTGACCATTGTCGAGGT |
|  | Reverse | TGTTGCCTGTACCACTTGTTTC |
| AHNAK2 F | Forward | TGTTGCCTGTACCACTTGTTTC |
|  | Reverse | GTGCAGAAACGGAAGATGACC |
| VEGF | Forward | GAGGAGCAGTTACGGTCTGTG |
|  | Reverse | TCCTTTCCTTAGCTGACACTTGT |
| E-cadherin | Forward | CGAGAGCTACACGTTCACGG |
|  | Reverse | GGGTGTCGAGGGAAAAATAGG |
| Vimentin | Forward | GACGCCATCAACACCGAGTT |
|  | Reverse | CTTTGTCGTTGGTTAGCTGGT |
| MMP2 | Forward | TACAGGATCATTGGCTACACACC |
|  | Reverse | GGTCACATCGCTCCAGACT |
| MMP9 | Forward | TGTACCGCTATGGTTACACTCG |
|  | Reverse | GGCAGGGACAGTTGCTTCT |
| ICAM1 | Forward | ATGCCCAGACATCTGTGTCC |
|  | Reverse | GGGGTCTCTATGCCCAACAA |
| ICAM2 | Forward | CGGATGAGAAGGTATTCGAGGT |
|  | Reverse | CACCCACTTCAGGCTGGTTAC |
| ITGB1 | Forward | CCTACTTCTGCACGATGTGATG |
|  | Reverse | CCTTTGCTACGGTTGGTTACATT |
| ITGB3 | Forward | GTGACCTGAAGGAGAATCTGC |
|  | Reverse | CCGGAGTGCAATCCTCTGG |
| CD44 | Forward | CTGCCGCTTTGCAGGTGTA |
|  | Reverse | CATTGTGGGCAAGGTGCTATT |
| P53 | Forward | CAGCACATGACGGAGGTTGT |
|  | Reverse | TCATCCAAATACTCCACACGC |
| SELP | Forward | ACTGCCAGAATCGCTACACAG |
|  | Reverse | CACCCATGTCCATGTCTTATTGT |
| SELE | Forward | AGAGTGGAGCCTGGTCTTACA |
|  | Reverse | CCTTTGCTGACAATAAGCACTGG |
