# Supplementary material for: Both SUMOylation and ubiquitination of TFE3 fusion protein regulated by androgen receptor are the potential target in the therapy of Xp11.2 translocation renal cell carcinoma
Source: Clin Transl Med. 2022 Apr 22;12(4):e797. doi: 10.1002/ctm2.797 (PMC9029019; doi:10.1002/ctm2.797)
Supplement: Supplementary file 13 — Supporting Information [file CTM2-12-e797-s008.docx]

**Supplement Table 5.** Primers used for luciferase reporter assay (Nhel, Hind III)

| Gene | Primer | Sequence |
| --- | --- | --- |
| ACCS | Forward | cgagctcttacgcgtgctagcTCACGAGGTCAGGAGTTCAAGAC |
|  | Reverse | cagtaccggaatgccaagcttCCACACGTGCCTGCAATATTT |
| RNF10 | Forward | cgagctcttacgcgtgctagcAGGAGATCAAGACCATCCTGGC |
|  | Reverse | cagtaccggaatgccaagcttGGTCTCGTGGTTTGTTTTTTGG |
| HIF-1A | Forward | cgagctcttacgcgtgctagcCCACGAGGCGAAGTCTGCT |
|  | Reverse | cagtaccggaatgccaagcttGCTCACGTGCTCGTCTGTGTT |
| MET | Forward | cgagctcttacgcgtgctagcGCGGTGCCCAAATCTCTCTA |
|  | Reverse | cagtaccggaatgccaagcttCATCGGCGCGCGCGGCCC |
| TRPM1 | Forward | cgagctcttacgcgtgctagcTGCCATGCCGTATCAGGAAA |
|  | Reverse | cagtaccggaatgccaagcttCCAGACTGAGGCGCTCG |
